# Biosorption of synthetic dyes by live and dried marine diatom *Phaeodactylum tricornutum* and freshwater diatom *Navicula cryptocephala var. veneta*

**DOI:** 10.3389/fmicb.2026.1891020

**Published:** 2026-09-30

**Authors:** Ceaira Howard, Tahira Tasneem Rahman, Mingyue Li, Kesler Hortin, Chiqian Zhang, Liyuan Hou

**Affiliations:** 1 Department of Civil and Environmental Engineering, Utah State University, Logan, UT, United States; 2 Utah Water Research Laboratory, Logan, UT, United States; 3 School of Resources and Environmental Engineering, Shandong University of Technology, Zibo, China; 4 Civil Engineering Program, College of Engineering and Computer Science, Arkansas State University, Jonesboro, AR, United States; 5 Department of Biological Engineering, Utah State University, Logan, UT, United States

**Keywords:** biosorption kinetics, dried and live diatoms, synthetic dyes, *Phaeodactylum tricornutum*, *Navicula cryptocephala var. veneta*

## Abstract

Diatom biomass has emerged as a promising biosorbent for removing dye pollutants from dye-bearing wastewater due to its low cost, sustainability, and abundant surface functional groups. Despite interest in algal biosorption, limited studies have compared live and dried biomass across different diatom species. The relative importance of species identity, biomass state, and operating conditions therefore remains unquantified. This study addresses these gaps by investigating the adsorption of three cationic dyes: crystal violet (CV), malachite green (MG), and basic fuchsin (BF), by two diatom species, *Phaeodactylum tricornutum* and *Navicula cryptocephala var. veneta*. The effects of dye concentration, pH, temperature, and biomass state were systematically evaluated. For dry cells, adsorption capacity increased with dye concentration due to enhanced mass transfer driving force. Maximum removal efficiencies by *P. tricornutum* reached 91.3% for CV, 88.6% for MG, and 85.9% for BF, corresponding to equilibrium adsorption capacities of 16.7, 36.6, and 26.6 mg/g, respectively, while *N. veneta* showed lower removal (13.8, 31.8, and 20.3 mg/g) and earlier saturation at higher concentrations. Neutral pH yielded optimal performance. However, MG and BF exhibited strong pH dependence because of surface charge effects and dye ionization, while CV was relatively insensitive to pH. Temperature effects were strongest for MG, smaller but significant for BF, and not significant for CV. Species and biomass state were the dominant factors controlling adsorption. *P. tricornutum* consistently outperformed *N. veneta* across all dyes and conditions tested. Dried biomass achieved significantly higher removal (CV = 70% to 91%, MG = 75% to 87%, BF = 61% to 84%) than live biomass (CV = 45% to 85%, MG = 70% to 82%, BF = 49% to 79%). This may be due to the drying process removing extracellular polymeric substances and membrane barriers, exposing reactive functional groups (e.g., -COO- and Si-O-) and promoting surface-controlled electrostatic interactions. Isotherm analysis indicated adsorption onto an energetically heterogeneous surface. The concentration range examined did not reach saturation in all systems, so maximum adsorption capacities are reported only where the data constrain them. These findings establish biomass state and species identity as the dominant controls on dye biosorption under the conditions tested. This work highlights dried marine and freshwater diatoms as sustainable biosorbents for broad-spectrum dye remediation.

## Highlights

- Both live and dried diatoms effectively adsorbed malachite green, crystal violet and basic fuchsin
- Dried cells of *P. tricornutum* and *N. veneta* exhibited higher adsorption capacities
- Isotherm analysis indicates adsorption onto energetically heterogeneous surfaces
- Live and dried diatoms have different major adsorption mechanisms

## 1 Introduction

Synthetic dyes are extensively used across a wide range of industries, including textile processing, dyeing and printing, tanning, paper and pulp manufacturing, cosmetics, food production, and pharmaceuticals (Shindhal et al., 2021). The global demand for these dyes has increased substantially over the past decades, with more than 10,000 commercially available dye types and an estimated annual production approaching 800,000 metric tons (Islam et al., 2023). Among these, the textile industry is one of the largest contributors to dye consumption and wastewater generation, releasing approximately 280,000 tons of dye-containing effluents into aquatic environments each year (Berradi et al., 2019). Alarmingly, nearly 80% of dye-laden wastewater is discharged into water bodies without adequate treatment or is reused directly for irrigation (Lin et al., 2023). The discharge of untreated synthetic dyes poses serious environmental and public health risks. Dye contamination leads to visible coloration of receiving waters, which reduces light penetration and disrupts photosynthetic activity in aquatic ecosystems. In addition, many synthetic dyes and their degradation products have carcinogenic, mutagenic, and teratogenic properties, posing toxicity risks to plants, animals, and humans (Lin et al., 2023; Mani et al., 2019). Consequently, the development of effective and sustainable treatment technologies for dye-containing wastewater has become an urgent priority.

Various physical, chemical, and biological approaches have been developed to treat dye-containing wastewater, including coagulation, biological degradation, advanced oxidation, electrochemical treatment, and membrane filtration (Al-Tohamy et al., 2022). Physicochemical treatments such as coagulation are widely used but produce large amounts of sludge requiring further management. Biological degradation using microorganisms or enzymes is environmentally friendly, yet it is typically time-consuming and relies on specific organisms. Physical separation methods, such as membrane filtration, can effectively remove dyes but are often constrained by high operational costs, membrane fouling, and energy demands (Prihartini Aryanti et al., 2025). Because of these limitations, adsorption has attracted increasing attention as an efficient alternative for dye removal across a wide range of dye concentrations. Biosorption has emerged as a promising strategy because it is cost-effective, environmentally friendly, and easy to operate. Biosorption uses natural or waste biomass as adsorbents, offering a sustainable approach for pollutant removal while reducing reliance on expensive conventional adsorbents such as activated carbon (Kumari et al., 2023; Youssef et al., 2023). Consequently, the development of low-cost biosorbents has become an active research area for wastewater treatment.

The three triphenylmethane dyes investigated in this study are of significant environmental concern due to their widespread industrial applications and potential toxicity (Upadhyay et al., 2023). These dyes include Crystal Violet (CV), malachite green (MG), and basic fuchsin (BF). CV is extensively used in biological staining, textiles, paints, and printing inks and has been associated with mutagenic, carcinogenic, and aquatic toxic effects (Mani and Bharagava, 2016). MG, widely applied in aquaculture, food, health, and textile industries, can form the persistent metabolite leucomalachite green and has been linked to carcinogenic, mutagenic, and reproductive toxicity (Srivastava et al., 2004). BF, primarily composed of pararosaniline derivatives, is used in textiles, paper, inks, paints, and biological staining, with pararosaniline classified as a potential carcinogen (Kowalska and Jeżewska, 2019). As triphenylmethane dyes possess complex aromatic structures and are resistant to biodegradation, conventional biological treatment processes often show limited removal efficiency (Przystaś et al., 2012), making adsorption an attractive polishing approach. Dye concentrations in textile effluents vary widely, ranging from 10–50 mg/L in general textile effluent to >100 mg/L in concentrated dyeing effluents (Yaseen and Scholz, 2019), highlighting the need for effective treatment strategies across diverse contamination levels. These dyes were selected because they share a common triphenylmethane chromophore and predominantly cationic character under the experimental conditions, while differing systematically in their amine substituents: CV carries three *N*,*N*-dimethylamino groups, MG contains two *N*,*N*-dimethylamino groups, and BF contains three unsubstituted primary amino groups (Supplementary Table S1). This structural series varies the degree of N-methylation, hydrogen-bonding capacity, molecular size, and basicity while maintaining a common chromophore, providing a comparative framework to evaluate the relative contributions of electrostatic interactions, hydrogen bonding, hydrophobic interactions, and π-π interactions during adsorption onto diatom biomass. The reported acid-base properties of these dyes also differ (Supplementary Table S1), resulting in distinct pH-dependent speciation behaviors that may influence their interactions with diatom surfaces across the investigated pH range.

Algae are promising biosorbents for wastewater treatment, particularly for the removal of dyes, due to their high adsorption capacity, wide availability, and the presence of cell wall polysaccharides containing functional groups such as hydroxyl, sulfate, and carboxyl groups that can effectively bind contaminants (González Fernández et al., 2023; Youssef et al., 2023). Additionally, many algal species can be cultivated using nutrient-rich wastewater, providing a sustainable and cost-effective source of biomass for pollutant removal. Among these algal taxa, diatoms are especially attractive because their silica-based cell walls, known as frustules, possess unique three-dimensional nanoporous structures that vary among species and provide exceptionally high specific surface areas (Rahman et al., 2024). These frustules also contain diverse functional groups, including silanol, amino, and carboxyl groups, as well as frustule associated proteins such as frustulins, that create multiple reactive binding sites for contaminants. Altogether, these structural and chemical characteristics make diatom biomass a promising, low-cost, eco-friendly, and renewable biosorbent for removing dyes from wastewater.

Despite the increasing interest in algal biosorption for wastewater treatment, several important research gaps remain. Most previous studies have focused primarily on green algal biomass or diatomite (sedimentary rock) (Abdullah Al-Dhabi and Arasu, 2022; Benhalima and Ferfera-Harrar, 2026; Bayramoglu et al., 2022; Rai et al., 2025). Although diatoms have unique silica-based frustule structures and highly reactive surfaces, studies investigating intact diatom biomass for dye adsorption remain comparatively limited (Howard et al., 2025) and have focused primarily on metal ion adsorption rather than dyes (Buhani et al., 2026; Wang et al., 2025; Yuan et al., 2025). Diatom frustules provide large specific surface areas and contain reactive functional groups such as -OH and -COOH that can interact with cationic dyes through electrostatic attraction, hydrogen bonding, and π-π interactions. Moreover, their nanoporous structures may further enhance adsorption through pore-filling mechanisms (Su et al., 2025). Unlike many other biosorbents, diatom frustules remain structurally intact after cell death, allowing dried biomass to retain strong biosorption capacity, whereas living cells may additionally remove pollutants through bioaccumulation and biotransformation. In addition, many biosorption studies rely on dried or chemically treated diatom biomass, paying limited attention to comparing the adsorption of live vs. dried cells, which may differ in surface accessibility, metabolic activity, and pollutant interaction mechanisms (González et al., 2024; Santaeufemia et al., 2021; Ashour et al., 2022). Current studies mainly focus on *Phaeodactylum tricornutum* (González et al., 2024; Santaeufemia et al., 2021; Yuan et al., 2025). Furthermore, the influence of species-specific structural characteristics on adsorption remains poorly understood, particularly among diatoms with varying frustule morphology and surface chemistry. Therefore, a systematic evaluation of different diatom species and physiological state (live vs. dried biomass) is needed to better understand the mechanisms governing dye adsorption and to optimize the application of diatom-based biosorbents for wastewater treatment.

This study aims to evaluate the biosorption performance of both live and dried biomass of a marine diatom *Phaeodactylum tricornutum* and a freshwater diatom *Navicula cryptocephala var. veneta* to remove representative cationic dyes. The central research question is how diatom species identity and biomass physiological state (live vs. dried) govern the biosorption of cationic dyes. To address this question, four specific aims were pursued: (1) to quantify the effects of operational parameters (biomass dosage, dye concentration, pH, and temperature) on dye removal and adsorption capacity; (2) to evaluate equilibrium adsorption behavior using isotherm models and assess adsorption characteristics under different dye–diatom combinations; (3) to compare the biosorption performance of live vs. dried biomass; and (4) to compare the two diatom species (*P. tricornutum* and *N. veneta*) and identify potential structural and chemical factors contributing to species-dependent differences. This study provides new insights into the use of diatom biomass to remove dyes by comparing the biosorption performance of live and dried cells from two diatom species with distinct frustule structures. The results demonstrate how operational conditions and biomass state influence adsorption performance and establish baseline adsorption capacities for untreated diatom biomass. While synthetic cationic dyes were selected as model contaminants, the adsorption mechanisms investigated in this study are broadly relevant to other positively charged pollutants. The comparative framework applied here may support future applications in the removal of heavy metals and other industrial contaminants from aquatic environments.

## 2 Materials and methods

### 2.1 Diatom biomass cultivation and preparation

The diatoms *P. tricornutum* (UTEX 646) and *N. veneta* (UTEX LB FD169) were purchased from the Culture Collection of Algae at the University of Texas at Austin (UTEX). The *P. tricornutum* was grown in an F/2 culture medium with an artificial seawater base, while the *N. veneta* was grown in an UTEX Modified COMBO medium derived from Guillard and Lorenzen WC medium (Howard et al., 2025; Utex, 2025a,b; Guillard and Lorenzen, 1972). The pH of the F/2 medium ranged between 7.8 and 8.0, while the Modified COMBO medium was adjusted to pH 7.8. Cultures were grown at 19 °C, 120–130 μE m^−2^ s^−1^ LED intensity at the flasks' surface for 12-h light/dark photoperiod and stirring at 90 rpm on a magnetic stir plate for *P. tricornutum*, and for *N. veneta*. To monitor diatom growth, 1.0 mL samples were taken from each active culture to measure the optical density at 680 nm (OD_680_) using a microplate reader (SpectraMax iD3, Molecular Devices). After reaching mid log-phase of growth (OD_680_ between 0.15 and 0.20), the cultures were used as seed cultures to inoculate a new growth medium (50 mL). The remaining 200 mL was left to reach the stationary growth phase for cell harvesting and dry cell preparation. To prepare diatom biomass for biosorption, cultures of *P. tricornutum* and *N. veneta* were grown photoautotrophically until late-log phase for dry cell harvesting and live cell harvesting. The cells were concentrated by centrifugation at 3,500 × g for 20 m, and cell pellets were washed sequentially with sterile phosphate buffered saline (PBS) and deionized (DI) water. For dry biomass, the cells were resuspended in 1 to 2 mL DI water and transferred to a pre-weighed 50 mL Falcon tube. The cells were left to dry in a 40 °C drying oven for 36 h. For live biomass, following the same centrifugation and wash steps, an additional two PBS washes and a final sterile DI water wash were performed following aseptic technique. Live cells were resuspended in sterile DI water before use. Prior to use, cell viability and morphology were verified by microscopy (ECHO Revolve R4, San Diego, CA).

### 2.2 Live and dry cell biosorption

Batch biosorption experiments were conducted to evaluate the dye removal performance of *P. tricornutum* and *N. veneta* using both live and dried biomass.

#### 2.2.1 Dyes and adsorption calculations

Dye removal efficiency (%) and adsorption capacity (mg/g) were determined for three cationic dyes: CV (ACS, 90+%, Alfa Aesar), MG (Thermo Scientific), and BF (Allied Chemical). Stock dye solutions were prepared at 460 μM in DI water and stored in amber glass bottles in darkness at room temperature to prevent light and temperature-induced degradation. The removal efficiency and adsorption capacity were calculated using Equations 1, 2:


$$\begin{array}{l} Removal\;efficiency\;\left(\%\right)=\frac{\left(C_{0}-C_{e}\right)}{C_{0}}\times 100 \end{array}$$ (1)

$$\begin{array}{l} Adsorption\;capacity\;\left(\frac{mg}{g}\right)=\frac{\left(C_{0}-C_{e}\right)\times V}{W} \end{array}$$ (2)

where *C*_0_ and *C*_*e*_ are the initial and equilibrium dye concentrations (mg/L), respectively; *V* is the solution volume (L); and *W* is the mass of diatom absorbent (*g*).

#### 2.2.2 Dried biomass batch experiments

Dry cell batch biosorption experiments were conducted to assess dye adsorption under varying biomass doses (0.1, 0.2, 0.4, and 0.8 mg/mL), dye concentration (1, 2.5, 5, 7.5, and 10 μM for CV and MG, 5, 7.5, 10, 15, and 20 μM for BF), temperatures (10, 20, and 30°C), and pH (unadjusted, 4, 6, and 7). The unadjusted pH values were 5.58 for CV, 4.65 for MG, and 5.88 for BF. Each parameter was assessed individually per dye in the order of biomass, dye concentration, temperature, and pH following the general batch biosorption experimental setup, including 20 mL working volume, 0.2 mg/mL biomass concentration, room temperature (20 °C), unadjusted pH, and continuous shaking, unless otherwise specified. Dried diatom biomass was added to 20 mL of 5 μM CV and MG (2.04 mg/L and 4.64 mg/L, respectively) and 10 μM BF (3.24 mg/L) at four different concentrations: 0.1, 0.2, 0.4, and 0.8 mg/mL (equivalent to 2, 4, 8, and 16 mg, respectively). The biomass concentration yielding the highest adsorption efficiency (i.e., the optimal biomass concentration) without saturation was selected for further tests. Using the optimal biomass concentration, a range of dye concentrations was tested: 1, 2.5, 5, 7.5, and 10 μM for CV and MG; and 5, 7.5, 10, 15, and 20 μM for BF. The concentration ranges differed between CV and MG (1 to 10 μM) and BF (5 to 20 μM) so that all three dyes fell within a comparable absorbance range on the microplate reader. These micromolar concentrations correspond to 0.4 to 4.1 mg/L for CV, 0.9 to 9.3 mg/L for MG, and 1.6 to 6.5 mg/L for BF. These ranges were selected to maintain absorbance within the reliable detection range of the analytical method while providing sufficient concentration gradients to evaluate adsorption behavior. The tested concentrations represent dilute dye-containing wastewater conditions rather than highly concentrated industrial dye-bath effluents. The dye concentration yielding the highest removal efficiency without saturation was selected for further tests. To determine the effects of pH on biosorption, dye stock solutions were adjusted to pH values of 4, 6, and 7 using 1 M HCl or 1 M NaOH and diluted to final concentrations of 10 μM for CV and MG, and 20 μM for BF. These concentrations were selected based on preliminary adsorption experiments that produced high removal efficiencies. Additionally, adsorption experiments were conducted at the natural, unadjusted pH of each dye solution because wastewater pH is not always controlled prior to treatment, and this condition represents adsorption without external chemical adjustment. The same unadjusted pH condition was also used for the live-cell experiments, allowing direct comparison between live and dried biomass under identical solution conditions. The adjusted and unadjusted dye solutions were added to the 50 mL amber glass tubes, followed by biomass addition. Negative controls at each pH were included, monitoring pH at the beginning and end of the batch experiment. The pH yielding the highest removal efficiency without saturation was selected for further tests. To study the influence of temperature on adsorption, three temperatures were chosen: 10, 20, and 30 °C, maintained using a temperature-controlled incubator (Fisher Scientific Isotemp). All other conditions remained consistent with the previous tests.

#### 2.2.3 Live biomass batch experiments

For live cell biosorption experiments, all experiments were performed in 50 mL amber glass bottles using 20 mL working volumes. Samples and blanks (no biomass) were shaken at room temperature (20 °C) using a Labquake Shaker Rotisserie (Thermo Scientific, Waltham, MA) with 1 mL aliquots collected at 5 m, 1, 2, and 4 h. Samples were immediately centrifuged at 3,500 × g for 2 m to pellet the biomass. The supernatants were analyzed for dye absorbance using a microplate reader (SpectraMax iD3, Molecular Devices) at dye-specific absorbance wavelengths: 592 nm for CV, 616 nm for MG, and 541 nm for BF. A standard curve was generated for each dye to correlate dye absorbance with concentration. All experiments were performed in biological duplicates with triplicate measurements.

The optimal parameters determined through dry cell biosorption were applied to live cell adsorption to conduct a single batch experiment with set biomass dose (0.2 mg/mL), dye concentration (10 μM for CV and MG and 20 μM for BF), temperature (20 °C), and pH (unadjusted). To determine the equivalent volume of live cells to represent the optimal dry biomass, a correlation between OD_680_ and dry biomass was established by diluting live cell samples (Saccardo et al., 2024), measuring their OD_680_, drying the cells, and recording the resultant dry masses. The dry biomass (mg/L) was then plotted against OD_680_ to generate a correlation per diatom species (see the Methods section in the Supplementary Figure S1). The live biosorption experiments followed the same preparation and execution as the dry cell experiments.

### 2.3 Kinetic and isotherm modeling

The data were fitted to non-linear Langmuir, Freundlich, and Sips isotherms (Equations 3, 4, and5, respectively) to determine the maximum adsorption capacity and to characterize the nature of the adsorption (Kumar et al., 2023):


$$\begin{array}{l} q_{e}=\frac{q_{\max}\times K_{L}\times C_{e}}{\left(1+K_{L}\times C_{e}\right)} \end{array}$$ (3)

$$\begin{array}{l} q_{e}=K_{f}\times {C_{e}}^{1/n} \end{array}$$ (4)

$$q_{e}=\frac{q_{\max}\times {\left(K_{s}\times C_{e}\right)}^{1/s}}{1+{\left(K_{s}\times C_{e}\right)}^{1/s}}$$ (5)

where *C*_*e*_ (mg/L) is the concentration of a dye in solution at equilibrium, q_e_ (mg/g) is the amount of a dye adsorbed at equilibrium per unit mass, q_max_ (mg/g) is the maximum sorption capacity, K_L_ (L/mg) is the Langmuir constant related to the affinity for the biosorbent, K_f_ [mg^1−(1/*n*)^ L^1/n^ g^−1^] is the Freundlich constant, n is the intensity of sorption, K_s_ (L/mg) is the Sips constant, and 1/s is the Sips exponent (Santaeufemia et al., 2021; Arshad et al., 2025).

Adsorption isotherms were fitted using non-linear least-squares regression of equilibrium adsorption capacity (q_e_, mg/g) as a function of equilibrium dye concentration (C_e_, mg/L). Because the Sips model contains an additional fitting parameter compared with the Langmuir and Freundlich models, model performance was evaluated using adjusted *R*^2^ in addition to *R*^2^. Model-derived maximum adsorption capacities were interpreted only when sufficiently constrained by the experimental data.

The time-resolved adsorption data from the dried-biomass experiments were fitted to the pseudo-first-order (Lagergren) and pseudo-second-order (Ho and McKay) models using non-linear regression (Equations 6, 7, respectively) (Ashour et al., 2022):


$$\begin{array}{l} q_{t}\;=\;q_{e}\left(1\;-\;e^{-k_{1}t}\right) \end{array}$$ (6)

$$\begin{array}{l} q_{t}\;=\;\frac{k_{2}q_{e}^{2}t}{1\;+\;k_{2}q_{e}t} \end{array}$$ (7)

where q_t_ (mg/g) is the adsorption capacity at time t (min), q_e_ (mg/g) is the calculated equilibrium adsorption capacity, k_1_ (min^−1^) is the pseudo-first-order rate constant, and k_2_ (g mg^−1^ min^−1^) is the pseudo-second-order rate constant.

Kinetic data were additionally evaluated using the Weber-Morris intraparticle diffusion model (Equation 8).


$$\begin{array}{l} q_{t}\;=\;k_{id}t^{1/2}+C_{d} \end{array}$$ (8)

where k_id_ (mg·g^−1^·min^−0.5^) is the intraparticle diffusion rate constant and C_d_ (mg/g) represents the boundary layer thickness. A non-zero intercept indicates that intraparticle diffusion is not the sole rate-limiting step and that external film diffusion or other mass-transfer processes may also contribute to adsorption (Ouettar et al., 2023).

Thermodynamic parameters were calculated from the equilibrium adsorption data collected at 10, 20, and 30 °C. The thermodynamic distribution coefficient (K_d_ = q_e_/C_e_, L/g) was converted to a dimensionless equilibrium constant by multiplying by the density of water. Standard Gibbs free energy (ΔG°) was calculated using ΔG° = –RT·lnK, while enthalpy (ΔH°) and entropy (ΔS°) were determined from linear regression of lnK vs. 1/T. Because thermodynamic parameters were derived from three temperature points, they were interpreted cautiously and reported with standard errors.

### 2.4 Diatom cell characterization

Morphological characterization was performed using field emission scanning electron microscopy (FE-SEM) coupled with energy dispersive X-ray spectroscopy (EDS). Cells were fixed in 2% buffered glutaraldehyde (1:10 to 1:20 by volume) at 4 °C overnight, deposited onto glass slides, and washed three times with 0.1 M HEPES buffer (5 m, gentle agitation) (Howard et al., 2025). Elemental composition was confirmed by EDS (Oxford, X-Max Detector) on copper conductive tape. Fourier-transform infrared (FTIR) spectroscopy (Thermo Scientific Nicolet 6,700) was used to characterize *P. tricornutum* and *N. veneta*. Washed cells were dried at 40 °C for 18 h, and spectra were collected (200 scans and 4 cm^−1^ resolution) over 4,000 to 675 cm^−1^ (Howard et al., 2025).

### 2.5 Data analysis

In the case of all growth and adsorption experiments, samples were collected in either duplicate or triplicate for each analysis. Samples taken in duplicate were to conserve active cultures or harvested biomass for continued growth and other studies. The data were analyzed with one-way ANOVA in Excel or two-way ANOVA with MATLAB. Unpaired Student's *t*-tests were used to determine significant differences between conditions. Differences were considered significant if *p* < 0.05. Standard deviation was calculated in each analysis to assess variability among biological and technical duplicates or triplicates. For each condition, abiotic controls (dye solution without biomass) were prepared at the same nominal concentration and pH, and measured at the same time points as the biomass-containing samples. The mean equilibrium concentration of these blanks was used as the initial concentration (C_0_) for calculating removal efficiency and adsorption capacity, so that reported removal is referenced against the blank and accounts for any non-biosorptive losses of dye (e.g., photodegradation, hydrolysis, or adsorption to container walls). The absorbance values were additionally corrected against a 0 μM (reagent) blank via the standard curve. Each condition was performed as two independent biological replicates, and each replicate was measured in triplicate (three absorbance readings). Results are reported as the mean of the biological replicates, and error bars represent the standard deviation between them. Differences between conditions, species, and biomass states were evaluated by one-way and two-way ANOVA and Student's *t*-tests described above.

## 3 Results and discussion

### 3.1 Effects of biomass dosage, initial dye concentration, pH, and temperature on dye adsorption

The two diatom species were characterized to provide the structural and surface-chemical context for interpreting the adsorption behavior discussed in subsequent sections. The morphology of both species has been previously characterized by FE-SEM (Howard et al., 2025). *P. tricornutum* has a fusiform morphology, with dimensions of approximately 16 μm in length and 3 μm in width, whereas *N. veneta* exhibits a smaller, more rounded morphology with dimensions of approximately 10 μm in length, 6 μm in width, and 2 μm in thickness. *P. tricornutum* lacks frustules with nanosized pores, while *N. veneta* frustules possess areolar pores approximately 100 nm in width and 100 to 400 nm in length (Figure 1). These pores were not observed to be fully penetrating and contained internal layers that may influence molecular accessibility. Elemental analysis by EDS reported in the same study revealed 58.0% carbon, 19.9% nitrogen, 18.8% oxygen, and 1.5% silicon for *P. tricornutum*, compared to 36.1% carbon, 6.7% nitrogen, 41.6% oxygen, and 16.6% silicon for *N. veneta*. These differences reflect the more organic-rich composition of *P. tricornutum* and the silicon-enriched frustule structure of *N. veneta*. FTIR spectra analysis confirmed common surface functional groups in both species, including hydroxyl groups (3,302 cm^−1^), C-H stretching (2,852 and 2,925 cm^−1^), amide-associated N-H bending (1,540 cm^−1^), C = O stretching (1,653 cm^−1^), and Si-O-Si stretching (1,057 cm^−1^) (Supplementary Figure S2). The Si-O-Si bending band at 795 cm^−1^ was present only in *N. veneta*, indicating differences in silica-associated surface chemistry. However, because FTIR was performed only before adsorption, these groups are considered potential adsorption-active sites rather than directly confirmed binding sites. Quantitative size distributions, surface area, pore structure, and post-adsorption characterization were not evaluated in this study.

**Figure 1:**
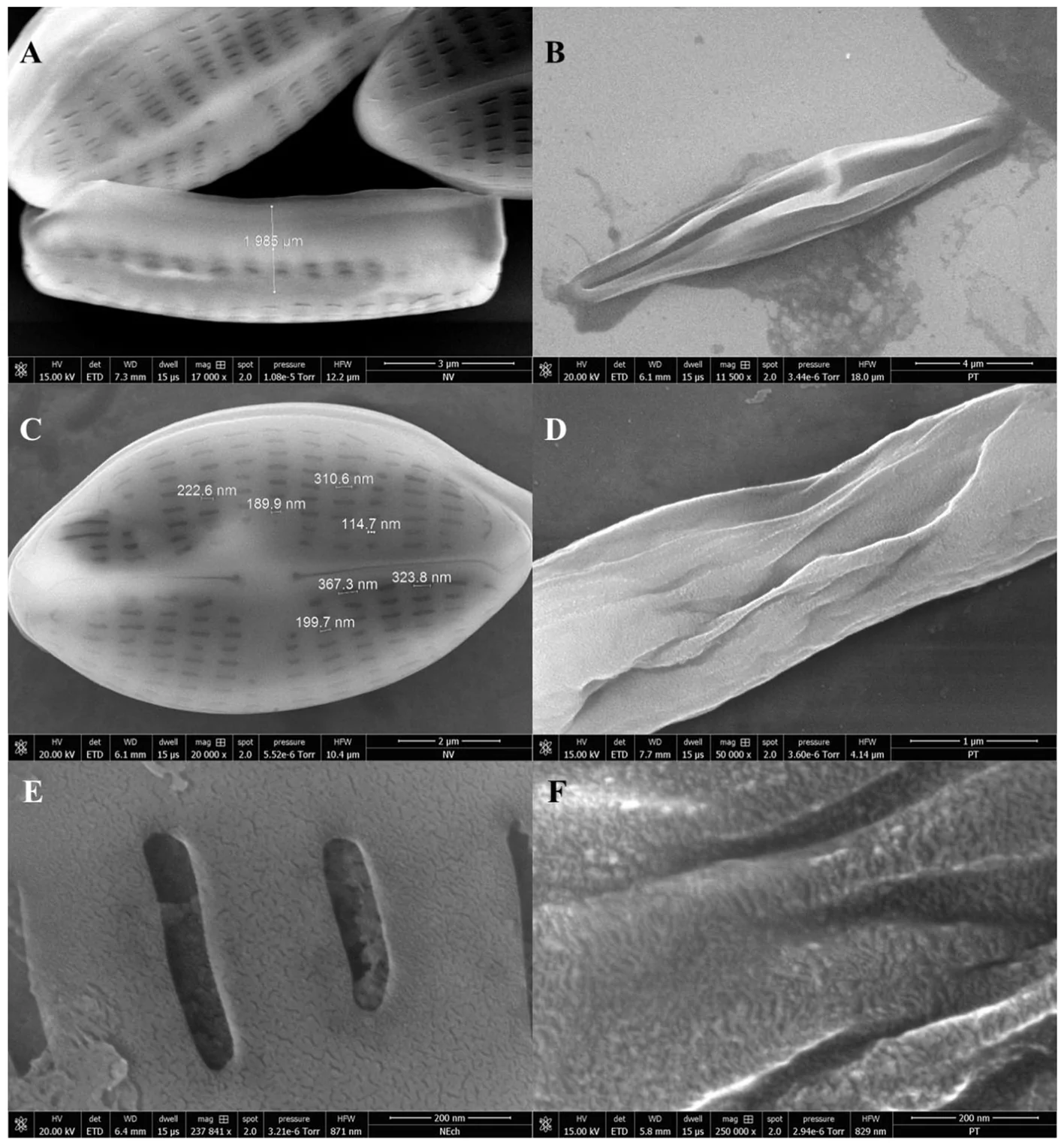
FE-SEM characterization of frustule morphology and surface microstructures of *N. veneta* (**A**, **C**, and **E**) and *P. tricornutum* (**B**, **D**, and **F**). Areolar pore dimensions measured from panel C are annotated.

Both *P. tricornutum* and *N. veneta* removed all three dyes effectively under the tested conditions. For each dye, adsorption capacity increased with initial dye concentration, while removal efficiency was highest near neutral pH and generally increased with temperature (Figures 2–4). The biosorption efficiency of diatoms is governed by the interplay of biomass dosage, initial dye concentration, solution pH and temperature (Bu et al., 2025). Initial trials identified the optimal biomass concentrations of *P. tricornutum* and *N. veneta* for effective removal of 5 μM CV and MG, and 10 μM BF (Supplementary Table S2). Higher adsorption capacity was achieved at lower biomass doses, which may be because adsorption sites remained less saturated and more accessible under these conditions (Thompson et al., 2020; Ashour et al., 2022). However, excessively low biomass concentrations may reduce the total dye removal efficiency due to insufficient available adsorbent. Then, adsorption performance was assessed across multiple experimental factors including dye concentration, solution pH, and temperature using the same biomass dose (0.2 mg/mL). The variation in dye concentrations (1, 2.5, 5, 7.5 and 10 μM for CV and MG; 5, 7.5, 10, 15, and 20 μM for BF at unadjusted pH and room temperature of 20 °C) showed effective removal by both diatom species, with performance varying by dye types and concentrations (Figure 2 and Supplementary Figure S3). A clear dose-dependent relationship between initial dye concentration and biosorption performance was observed (Supplementary Table S3). For both *P. tricornutum* and *N. veneta*, an increase in the initial concentration of CV, MG, and BF led to higher absolute adsorption capacities (mg/g). This is attributed to the increased driving force provided by higher dye concentrations, which effectively overcomes the resistance to mass transfer between the bulk liquid phase and the solid diatom surface (Long et al., 2024). This suggests that adsorption typically occurs in two stages: a rapid initial phase where dye molecules occupy vacant surface sites, followed by a slower phase as these sites become saturated. At higher concentrations, the increased number of dye molecules promotes diffusion into the nanoporous architecture of the frustules, allowing for higher maximum loading (Golubeva et al., 2023b; Howard et al., 2025). However, there is a physical limit. For instance, while *P. tricornutum* achieved its maximum removal (91.28%) for CV at 10 μM, *N. veneta* began to show a decline in removal at higher dye concentrations (56.79% for BF at 15 μM), indicating that its active sites were more easily saturated (Supplementary Table S3, Figure 2 and Supplementary Figure S3). The adsorption efficiencies were evaluated across a range of pH conditions (unadjusted, 4, 6, and 7) to assess how solution chemistry influences biosorption (Supplementary Table S4). Adsorption capacity and removal efficiency were monitored over time for each dye (Figure 3 and Supplementary Figure S4). The two-way ANOVA analysis for dye pH vs. diatom species showed dye pH significantly affected adsorption for MG and BF (*p* < 0.05, Table 3) but not for CV. Solution pH exerted a clear and dye-dependent influence on adsorption. For CV, adsorption capacity showed relatively minor variation across pH conditions, with slightly enhanced performance observed near neutral pH (pH 6). Furthermore, CV has an extremely low pK_a_ (approximately 0.8) (Ashour et al., 2022), meaning it remains positively charged across nearly all experimental pH conditions, which contributes to its relatively stable adsorption performance over the tested pH range. The insensitivity to pH suggests that CV adsorption is not governed by electrostatic interactions alone. The adsorption may also involve non-electrostatic mechanisms such as hydrophobic interactions or π-π stacking, such as the formation of surface hydrogen bonds between the nitrogen atoms of the dye molecule and the hydroxyl (silanol) groups on the diatom surface (Al-Ghouti et al., 2003). By contrast, MG exhibited a pronounced dependence on pH, with significantly lower adsorption under acidic conditions (pH 4: 78.53% ± 3.73% and 68.29% ± 6.47% for *P. tricornutum* and *N. veneta*, respectively) and progressively higher capacities toward neutral and slightly alkaline conditions (pH 6 to 7). Similarly, BF adsorption was consistently suppressed at low pH and improved under neutral conditions, although its response to pH was less pronounced than MG. These patterns align with the ANOVA results (Supplementary Table S5) and indicate that the adsorption of MG and BF is more sensitive to changes in surface charge and dye speciation. The surface charge of diatom-based adsorbents is highly sensitive to the pH of the medium because of the presence of hydroxyl (silanol) groups, as the point of zero charge (pH_*pzc*_) of diatom surface is between 4.9 and 6.0 (Cely Bautista et al., 2022; Khraisheh et al., 2005; Golubeva et al., 2023a). Under acidic conditions, increased protonation likely reduces the availability of negatively charged binding sites and introduces competition between protons and dye molecules, leading to reduced adsorption efficiency (Golubeva et al., 2023a). Also, at acidic pH, both the dye molecules and the adsorbent surface are positively charged, leading to electrostatic repulsion that inhibits adsorption. MG is more sensitive to pH than the other two dyes as MG has an ionization constant (pK_a_) of 6.90. At pH 4.0, it is 100% ionized, but by pH 6.9, it is half ionized. This significant shift in the dye's molecular state near neutral pH makes its interaction with the surface highly sensitive to pH changes. This allows the evaluation of biosorption under realistic wastewater conditions, where pH may not always be controlled. The unadjusted pH was selected for further studies because it consistently supported high adsorption performance across both diatom species and dye types, making it an optimal condition.

**Figure 2:**
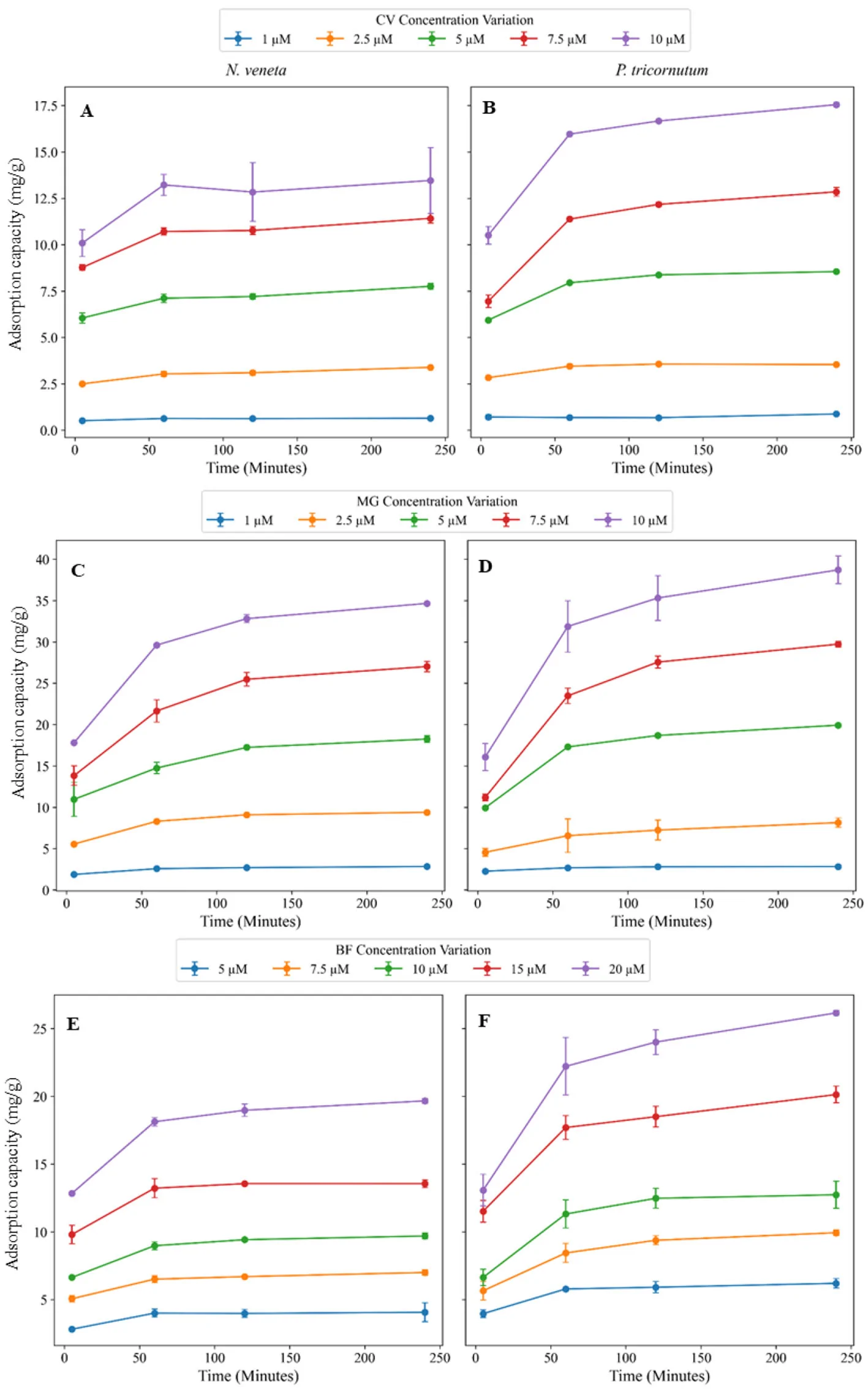
Adsorption capacity (mg/g) of CV, MG, and BF by dried *N. veneta* (**A**, **C**, and **E**) and *P. tricornutum* (**B**, **D**, and **F**) under varying initial dye concentrations. CV and MG were tested at 1–10 μM, and BF was tested at 5–20 μM. Experiments were conducted at 20 °C with an initial biomass concentration of 0.2 mg/mL. Error bars represent the standard deviation of two biological replicates.

**Figure 3:**
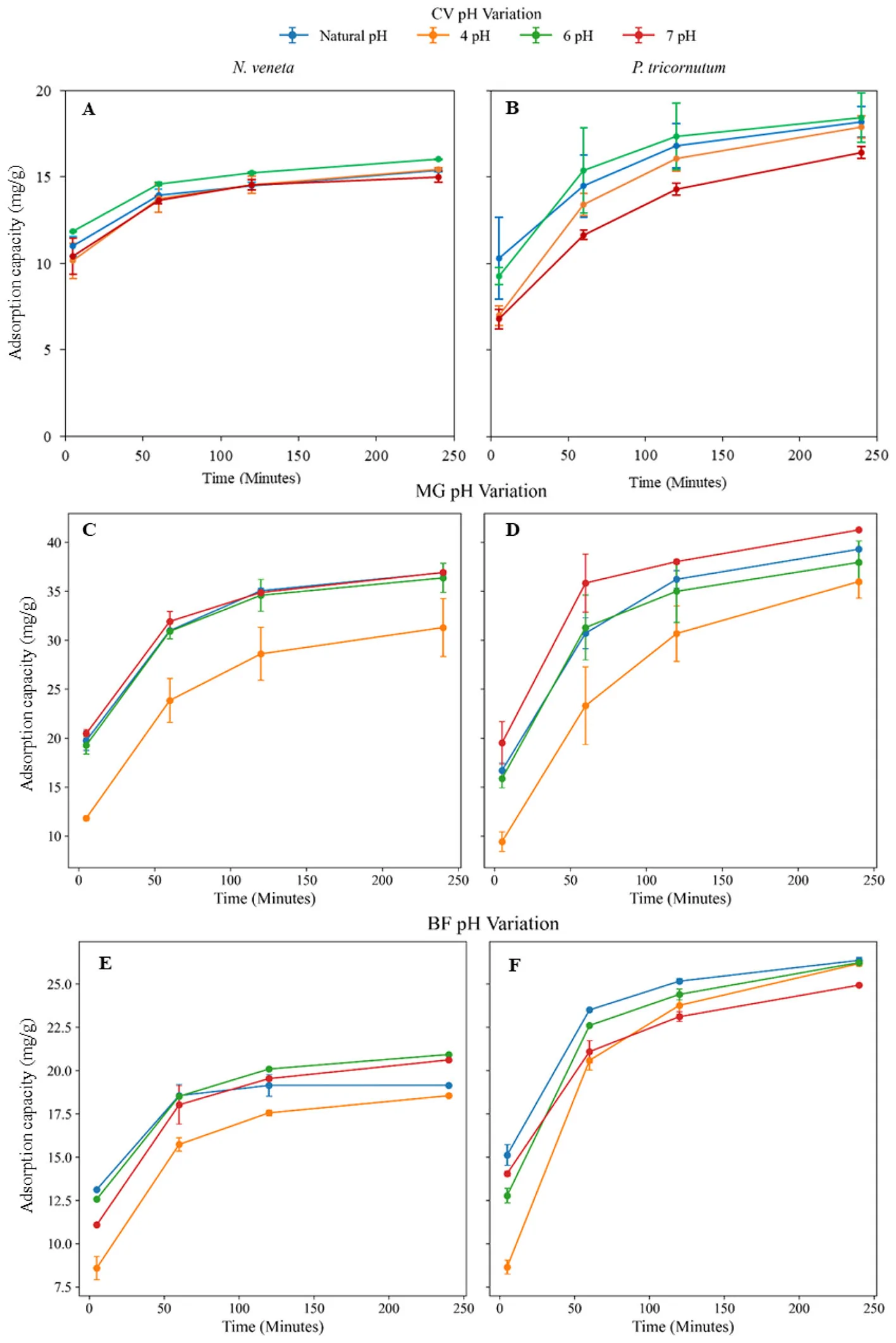
pH-dependent adsorption capacity of CV, MG, and BF by dried *N. veneta* (**A**, **C** and **E**) and *P. tricornutum* (**B**, **D**, and **F**) at an initial biomass concentration of 0.2 mg/mL. Experiments were conducted at 20 °C with initial dye concentrations of 10 μM for CV and MG, and 20 μM for BF. Error bars represent the standard deviation of two biological replicates.

**Figure 4:**
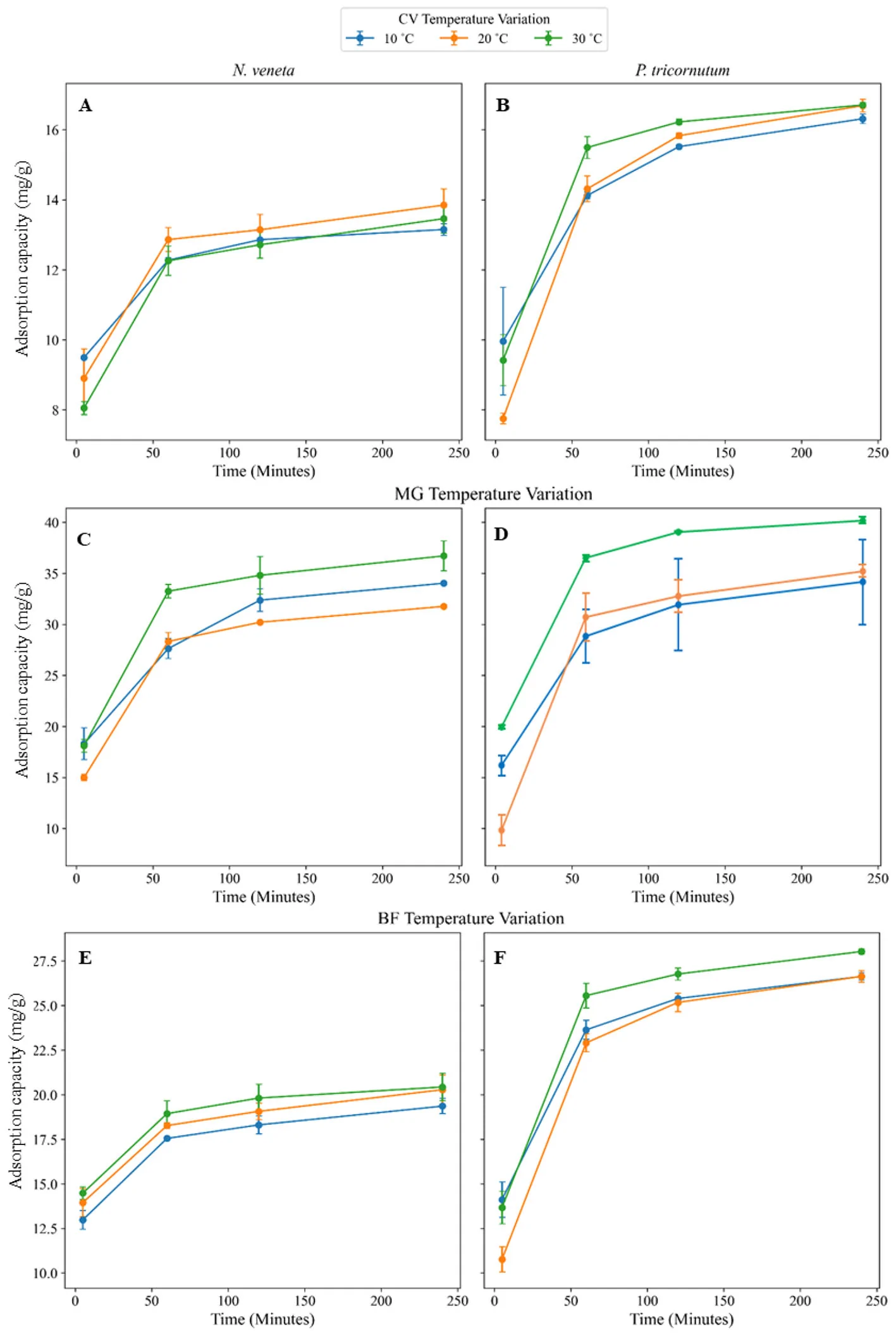
Temperature-dependent adsorption capacity of CV, MG, and BF by dried *N. veneta* (**A**, **C**, and **E**) and *P. tricornutum* (**B**, **D**, and **F**) at an initial biomass concentration of 0.2 mg/mL. Experiments were conducted at 10, 20, and 30 °C with initial dye concentrations of 10 μM for CV and MG, and 20 μM for BF. Error bars represent the standard deviation of two biological replicates.

Temperature had a measurable effect on dye removal and adsorption capacity across both diatom species and dye types (Figure 4, Supplementary Figure S5 and Supplementary Table S6). Adsorption capacity increased with temperature for all dyes and both diatom species, with the most pronounced enhancement observed for MG and BF (*p* < 0.05, Supplementary Table S5). For instance, MG removal by *P. tricornutum* increased from 79.1% ± 9.8% at 10 °C to 92.2% ± 0.9% at 30 °C, with a similar but weaker trend for BF. This suggests that the adsorption of MG and BF is more diffusion- or activation-controlled, potentially reflecting differences in molecular structures, sizes, and interaction with surface functional groups. For CV, adsorption capacity and removal efficiency remained stable across temperatures, with slight increases from 10 to 30 °C (e.g., ~89 to 92% removal for *P. tricornutum* and ~72 to 76% for *N. veneta*). Adsorption capacity showed minimal variation (e.g., ~16.3 to 16.7 mg/g by *P. tricornutum*), and the effect of temperature on CV was not statistically significant. MG and BF are more sensitive to temperature because their uptake may involve diffusion-limited and endothermic processes that benefit from increased thermal energy (Dubey et al., 2024). In short, dye concentration set the adsorption driving force, whereas the effects of pH and temperature on removal were dye-dependent, with CV being largely insensitive to either factor, and MG and BF responding strongly.

### 3.2 Adsorption kinetics, isotherms, and thermodynamics

Beyond the equilibrium removal, adsorption kinetics were evaluated by fitting pseudo-first-order and pseudo-second-order models to the time-resolved data (Table 1 and Supplementary Figure S6). For all three dyes and both species, the pseudo-second-order model provided the better fit (*R*^2^ ≥ 0.95), with calculated adsorption capacities closely matching the experimental values. This agrees with previous studies of CV adsorption by marine diatoms, in which pseudo-second-order kinetics also provided the best fit (Ashour et al., 2022). Because the models were fitted using only four time points, the estimated rate constants should be interpreted as indicative rather than definitive.

**Table 1:** Pseudo-first-order, pseudo-second-order, and intraparticle diffusion kinetic parameters for the adsorption of CV, MG, and BF by dried *P. tricornutum* and *N. veneta* at 20 °C.

Dye	Species	q_e, exp_ (mg/g)	Pseudo-first-order			Pseudo-second-order			Intraparticle diffusion		
			q_e, cal_	k_1_ (min^−1^)	*R* ^2^	q_e, cal_	k_2_ (g·mg^−1^ ·min^−1^)	*R* ^2^	k_id_ (mg·g^−1^·min^−0.5^)	C_d_ (mg/g)	*R* ^2^
CV	*P. tricornutum*	16.69	15.61	0.137	0.941	16.54	0.0102	0.982	0.676	7.49	0.866
CV	*N. veneta*	13.85	13.28	0.222	0.965	13.64	0.0272	0.986	0.365	8.86	0.832
MG	*P. tricornutum*	36.55	34.58	0.058	0.983	38.77	0.00175	0.998	2.003	9.99	0.799
MG	*N. veneta*	31.76	30.10	0.138	0.967	31.80	0.00548	0.994	1.248	15.21	0.811
BF	*P. tricornutum*	26.63	24.92	0.112	0.956	26.82	0.00479	0.992	1.195	10.49	0.848
BF	*N. veneta*	20.28	19.20	0.259	0.910	19.62	0.0245	0.946	0.472	13.60	0.912

Experimental data and model fits are shown in Supplementary Figure S6.

The Weber-Morris intraparticle diffusion model was also fitted to the kinetic data (Table 1 and Supplementary Figure S7). The intraparticle diffusion rate constant was consistently higher for *P. tricornutum* than for *N. veneta* across all three dyes (0.676 vs. 0.365 for CV, 2.003 vs. 1.248 for MG, and 1.195 vs. 0.472 mg·g^−1^·min^−0.5^ for BF). In all systems, the intercept was substantially greater than zero, from 27 to 67% of the final capacity, and was statistically different from zero for CV and BF on *N. veneta* (*p* = 0.018 and 0.006, respectively). These results indicate that intraparticle diffusion was not the sole rate-limiting step and that boundary-layer effects were most pronounced for *N. veneta*. However, because only four time points were available, distinct diffusion regimes could not be resolved, and the resulting fits should therefore be interpreted as single-region approximations.

To better understand adsorption mechanisms and evaluate the biosorption potential of *P. tricornutum* and *N. veneta*, we applied three isotherm models (Langmuir, Freundlich, and Sips) to equilibrium adsorption data for CV, MG, and BF (Figure 5 and Table 2). Among these, the Sips model provided the best fit for CV and MG adsorption on *N. veneta*, whereas the Freundlich model performed better for BF adsorption. In contrast, the Langmuir model yielded physically unrealistic maximum adsorption capacities for most systems. These models describe how dye molecules distribute between the liquid phase and the dry cell biomass at equilibrium, characterizing surface interactions, binding strengths, and adsorption capacities. The Langmuir isotherm assumes monolayer adsorption onto a homogeneous surface with uniform binding energies and no interaction between adsorbed molecules (Shimizu and Matubayasi, 2023). While commonly applied in ideal adsorption systems, the Langmuir model did not yield a physically meaningful maximum adsorption capacity for most systems, with fitted values exceeding the highest measured capacities by two to three orders of magnitude. These values are reported in Table 2 for completeness but represent extrapolation artifacts and should not be interpreted as experimentally supported maximum adsorption capacities. By contrast, the Freundlich isotherm, an empirical model accounting for heterogeneous surfaces and multilayer adsorption, provided improved fits across most systems (*R*^2^ up to 0.98). The fitted Freundlich exponents ranged from 0.5 to 1.42. Values above unity, obtained for CV and MG adsorption on *N. veneta*, indicate favorable adsorption, whereas values below unity for *P. tricornutum* and BF adsorption by *N. veneta* are consistent with isotherms lacking a clear plateau and equilibrium data that did not reach saturation.

**Figure 5:**
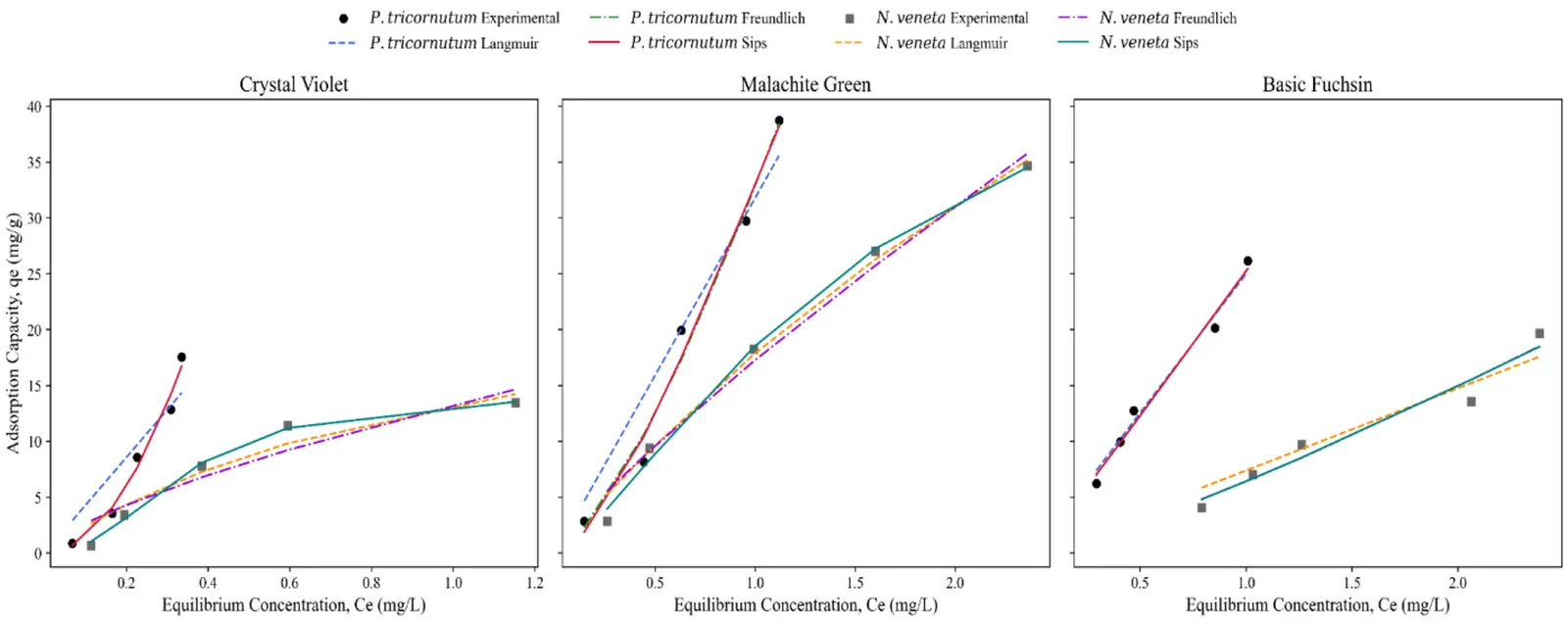
Isotherm plots (Langmuir, Freundlich, and Sips) for CV (**A**), MG (**B**), and BF (**C**) by N. veneta and P. tricornutum.

**Table 2:** Isotherm model parameters (Langmuir, Freundlich, and Sips) for the adsorption of CV, MG, and BF by dried *P. tricornutum* (Pt) and *N. veneta* (Nv).

Dye	Species	Langmuir			adj R^2^	Freundlich			adj R^2^	Sips				adj R^2^
		q_max_ (mg/g)	K_L_ (L/mg)	R^2^		K_f_ (mg/g)	n	R^2^		q_max_ (mg/g)	K_s_ (L/mg)	s	R^2^	
CV	Pt	5,352.8	0.01	0.85	0.693	149.0	0.5	0.98	0.961	1315.4	0.34	2.01	0.98	0.921
	Nv	27.4	0.94	0.93	0.860	13.3	1.42	0.89	0.776	14.4	2.89	2.31	1	0.985
MG	Pt	22,611.7	0	0.94	0.889	32.8	0.72	0.98	0.967	187.6	0.38	1.59	0.98	0.936
	Nv	121.32	0.17	0.99	0.980	17.3	1.18	0.98	0.966	54.8	0.62	1.4	1	0.985
BF	Pt	4,452.0	0.01	0.98	0.964	25.2	0.96	0.98	0.966	543.2	0.06	1.08	0.98	0.933
	Nv	27,929.4	0	0.93	0.855	6.4	0.82	0.95	0.903	401.2	0.04	1.26	0.95	0.805

However, the absence of a defined maximum adsorption capacity limits its predictive value for process design and capacity estimation. The Sips isotherm, which integrates Langmuir and Freundlich behavior, provided the closest fit for CV and MG adsorption by *N. veneta*, where the fitted maximum capacities were consistent with the measured data. In contrast, for the remaining systems, the additional Sips parameter did not provide sufficient improvement in model performance when model complexity was considered, largely because the experimental data did not approach a clear saturation plateau. Consequently, the regressions could not reliably constrain a unique maximum adsorption capacity. A similar limitation was evident for the Langmuir model, for which fitted maximum capacities exceeded the highest experimentally measured capacities by factors of 170 to 1,421 for CV, MG and BF adsorption by *P. tricornutum* and for BF on *N. veneta*, with the corresponding affinity constants approaching zero. These values therefore represent poorly constrained extrapolations rather than physically meaningful adsorption capacities. Reliable maximum capacities are therefore obtained only for CV and MG adsorption by *N. veneta*, with Sips-derived *q*_max_ values of 14.4 and 54.8 mg/g, respectively; accordingly, only these values are interpreted further. The heterogeneity index (s) ranged from 1.08 to 2.31, suggesting moderate heterogeneity in the binding environment and the possibility of multilayer adsorption.

The limited saturation observed in the isotherm analysis also provides important context for comparing the adsorption capacities measured here with those reported for other diatomaceous adsorbents. The capacities obtained in this study are lower than many literature values, largely because the experiments were conducted over a relatively low dye concentration range. At the biomass dose used (0.2 mg/mL), the maximum uptake attainable under each experimental condition is constrained by the amount of dye initially available. For the measured initial concentrations of 8.957 μM CV, 8.896 μM MG, and 19.152 μM BF, the corresponding theoretical uptake limits were 18.27, 41.28, and 31.03 mg/g, respectively. At 20 °C, the measured capacities reached 91.4, 88.6, and 85.8% of these limits, indicating that most of the available dye had been removed from solution. Thus, the measured capacities were primarily constrained by dye availability and should not be interpreted as the intrinsic maximum adsorption capacities of the biomass. In addition, the biomass was used without chemical or thermal activation; therefore, the values reported here represent adsorption performance of intact, minimally processed diatom biomass under low-concentration conditions.

Although the concentration range limited determination of intrinsic maximum adsorption capacities, the temperature-dependent experiments provide complementary information on the thermodynamic favorability of adsorption (Supplementary Table S7). ΔG° was negative for all dye–species combinations across the three tested temperatures (−21.2 to −27.7 kJ/mol), indicating spontaneous adsorption, whereas positive ΔH° values indicated an endothermic process. The strongest temperature dependence was observed for MG adsorption by *P. tricornutum*, which exhibited the largest ΔH° (+40.57 ± 5.73 kJ/mol, *R*^2^ = 0.980), followed by MG adsorption on *N. veneta* (+13.05 ± 5.47 kJ/mol) and CV adsorption by *P. tricornutum* (+12.90 ± 3.43 kJ/mol). In contrast, the Van't Hoff regressions for CV and BF adsorption by *N. veneta* were weak (*R*^2^ = 0.357 and 0.370, respectively) because adsorption changed only slightly across the tested temperature range, resulting in uncertainties that approached or exceeded the estimated ΔH° values. These thermodynamic parameters are therefore reported for completeness but are not interpreted further. Overall, the isotherm analysis indicates that adsorption of the synthetic dyes onto diatom biomass is governed by heterogeneous surface interactions rather than monolayer adsorption. The improved performance of the Freundlich and, for selected systems, Sips models suggests a distribution of adsorption sites with different affinities, while the lack of a well-defined saturation plateau for several systems limits reliable estimation of maximum adsorption capacity. This heterogeneous adsorption behavior is consistent with the diverse surface functional groups identified by FTIR and the structurally complex, composite organic-inorganic nature of diatom biomass and frustules.

### 3.3 Effects of biomass state on dye adsorption

Because drying alters both the physical accessibility of the frustule and the viability of the cell, the two biomass states were compared directly under identical conditions. This isolates the contribution of the biological barriers present in living cells from the intrinsic adsorption properties of the frustule. Dried biomass removed more dye than live biomass for both species and all three dyes, though live cells followed the same overall trends (dried: CV 70–91%, MG 75–87%, BF 61–84%; live: CV 45–85%, MG 70–82%, BF 49–79%) (Figure 6). *P. tricornutum* consistently outperformed *N. veneta* across all dyes, removing 85.00% ± 1.36% of CV, 82.80% ± 0.10% of MG, and 78.80% ± 2.59% of BF. By contrast, *N. veneta* showed substantially lower removal of CV (45.36% ± 0.88%) and BF (48.69% ± 3.10%), while MG removal (70.10% ± 5.29%) approached that of *P. tricornutum*. Adsorption capacity followed a similar trend. *P. tricornutum* demonstrated higher uptake per unit biomass (e.g., 36.51 ± 0.04 mg/g for MG), whereas *N. veneta* exhibited lower capacities, particularly for CV and BF.

**Figure 6:**
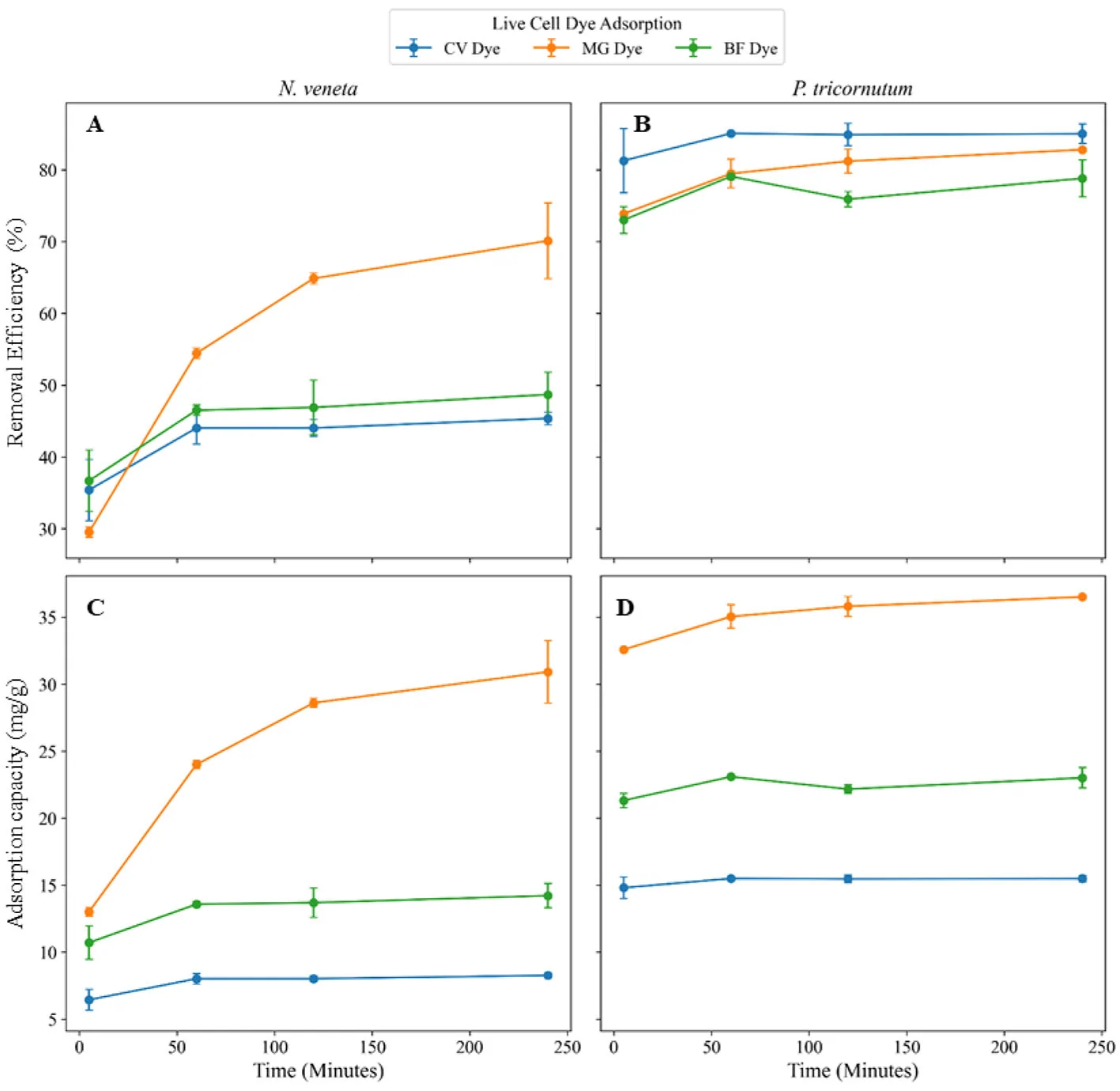
Removal efficiency (%) and adsorption capacity (mg/g) of CV (10 μM), MG (10 μM), and BF (20 μM) by live *N. veneta* (**A** and **C**) and *P. tricornutum* (**B** and **D**) as a function of contact time. Experiments were conducted at the unadjusted (natural) pH of each dye solution (CV = 5.58, MG = 4.65, and BF = 5.88) and 20 °C, with an initial biomass concentration of 0.2 mg/mL. Error bars represent the standard deviation of two biological replicates.

Compared with dry biomass under optimized conditions (20 °C and unadjusted pH), live cells showed moderately reduced adsorption performance. Importantly, the comparison between live and dried biomass, and between the marine and freshwater species, was performed under consistent experimental conditions. In each case, the same DI water, dye concentration, unadjusted pH, temperature (20 °C), and biomass dose (0.2 mg/mL) were used, rather than the respective marine or freshwater growth media. Because all biosorption experiments were carried out in DI water, differences in the ionic strength or pH of the two growth media did not contribute to the observed differences, which can instead be attributed to biomass state (live vs. dried) and species-specific characteristics. Live cells may also take up dye across the cell wall in addition to surface biosorption. Given the short contact times used here ( ≤ 4 h), surface biosorption is expected to dominate, although the present data cannot fully separate these contributions. This difference can be attributed to the predominantly passive, surface-mediated nature of biosorption. In living diatoms (Figure 7), the extracellular polymeric substance (EPS) layer and intact cell membranes act as physical barriers that limit the accessibility of dye molecules to reactive binding sites on the silica frustule (Li et al., 2021). Drying is expected to disrupt these biological barriers and increase access to the underlying frustule surface. Previous work on *P. tricornutum* attributed differences between living and dried biomass to loss of cell integrity, which facilitates dye entry, with the magnitude of this effect depending on dye toxicity rather than changes in surface chemistry (Santaeufemia et al., 2021). However, because the biomass surface was not characterized after drying or adsorption in the present study, this interpretation represents a plausible explanation for the observed differences rather than a directly demonstrated mechanism. Although living cells can contribute to dye removal through active processes such as bioaccumulation and biotransformation, biosorption is generally the dominant mechanism during short-term adsorption experiments because it occurs rapidly at the cell surface, whereas intracellular uptake and metabolic transformation require longer contact times (Yilmazer Keskin et al., 2025). A previous study supports this distinction: dried *P. tricornutum* biomass exhibited higher removal efficiency for methylene blue (MB) at concentrations up to 10 mg/L of MB (31.3 μM), likely due to reduced membrane resistance and improved accessibility of adsorption-active sites (Santaeufemia et al., 2021). In contrast, for safranin, living *P. tricornutum* cells showed improved performance at low dye concentrations (<10 mg/L, ~28.5 μM), suggesting that lower toxicity allows metabolic uptake to complement surface adsorption.

**Figure 7:**
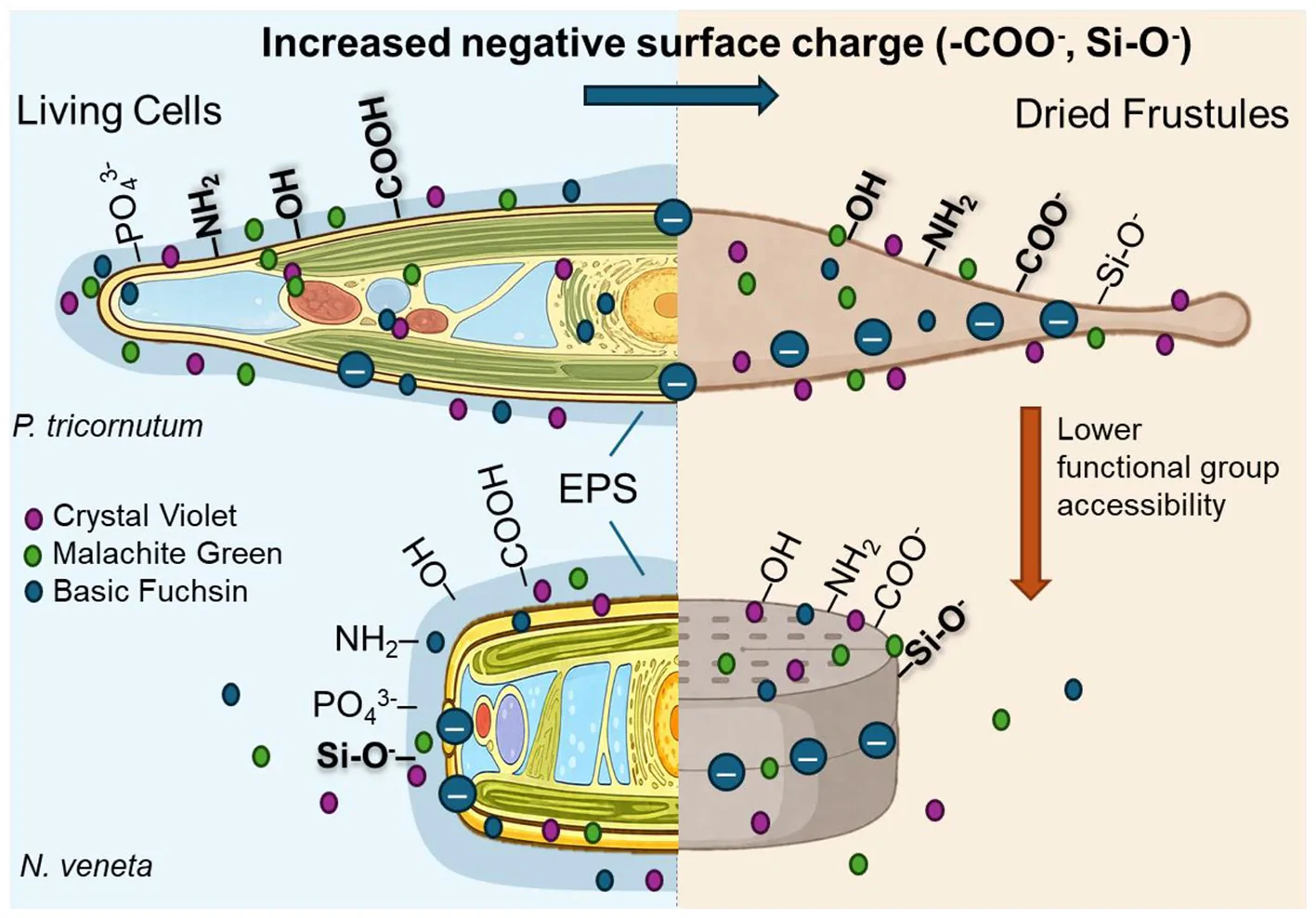
Conceptual schematic of the proposed dye adsorption mechanisms in living cells and dried frustules of *P. tricornutum* and *N. veneta*. The illustrated mechanisms represent candidate pathways based on the functional groups detected by FTIR before adsorption and were not confirmed by post-adsorption characterization. Abundant surface functional groups (e.g., -COOH, -NH_2_, -OH) are highlighted in bold.

Structural differences between the two species further contribute to their adsorption behavior. To better interpret the influence of frustule morphology on adsorption behavior, it is important to distinguish between adsorption capacity and adsorption kinetics. Adsorption capacity reflects the maximum amount of dye retained at equilibrium, whereas adsorption kinetics describes the rate at which adsorption occurs. The porous frustule architecture of *N. veneta* may provide additional internal binding sites and potentially enhance adsorption capacity (Figure 7). However, this structural complexity may also introduce internal diffusion resistance, slowing dye transport into micro- and nanoporous regions (Howard et al., 2025; Cantonati et al., 2016). In contrast, *P. tricornutum* likely favors faster surface-dominated adsorption with lower mass transfer resistance. (Howard et al., 2025; Chuberre et al., 2022). Therefore, structural complexity may slow adsorption kinetics without necessarily reducing the total adsorption capacity. Overall, these results indicate that dry biomass enhances adsorption by removing biological barriers and increasing access to active binding sites, while species-specific structural characteristics influence the balance between surface accessibility and diffusion-controlled uptake. These findings are consistent with our previous study using second harmonic scattering spectroscopy, which demonstrate that dye uptake in live diatoms is governed by both surface interactions and transport limitations (Howard et al., 2025). In the present study, drying likely enhanced adsorption by removing biological barriers such as EPS layers and membranes, thereby improving accessibility to adsorption-active surface sites. Thus, drying enhances adsorption by removing the EPS and membrane barriers that limit dye access to the frustule, while species-specific frustule structure governs the balance between surface accessibility and diffusion-controlled uptake.

### 3.4 Species-specific adsorption performance and proposed mechanisms

Having established the effects of operating parameters and biomass state, we next compare the two species and examine the structural and chemical characteristics that may contribute to their differences in adsorption performance. *P. tricornutum* consistently outperformed *N. veneta* across all dyes and conditions, exceeding 80% removal for most dyes and reaching 91.28% ± 0.57% for CV at 10 μM, whereas *N. veneta* showed lower and more variable removal (e.g., BF fell to 56.79% ± 1.17%) (Figures 2–4 and Table 3). The effect of species on biosorption performance was consistently significant across all experimental conditions, indicating that the intrinsic differences between *P. tricornutum* and *N. veneta* play an important role in adsorption behavior. Two-way ANOVA confirmed that species had a statistically significant main effect (*p* < 0.05) for dye type, concentration, pH, and temperature conditions (Table 3), demonstrating a strong contribution of species identity to adsorption. However, interactions between species and environmental factors were generally insignificant for pH and temperature (*p* > 0.05). Therefore, while the overall adsorption capacity differs between species, their responses to changes in these environmental conditions follow similar trends.

**Table 3:** Two-way ANOVA results for the effects of dye type, initial dye concentration, pH, and temperature on dye adsorption by two diatom species.

Comparison	Main factor (*p*)	Species (*p*)	Interaction (*p*)
Dye type	<0.001^*^	0.006^*^	0.007^*^
Concentration (CV)	<0.001^*^	<0.001^*^	0.004^*^
Concentration (MG)	<0.001^*^	<0.001^*^	<0.001^*^
Concentration (BF)	<0.001^*^	<0.001^*^	<0.001^*^
pH (CV)	0.059	<0.001^*^	0.518
pH (MG)	0.005^*^	0.003^*^	0.457
pH (BF)	<0.001^*^	<0.001^*^	<0.001^*^
Temperature (CV)	0.084	<0.001^*^	0.577
Temperature (MG)	0.017^*^	0.014^*^	0.371
Temperature (BF)	0.041^*^	<0.001^*^	0.294

Values are *p*-values; ^*^ indicates *p* < 0.05. (Full statistics, including *F*-values, are provided in Supplementary Table S5). Separate analyses evaluated each experimental factor, diatom species, and their interaction. Concentration, pH, and temperature were analyzed separately for each dye.

Dye concentration significantly influenced adsorption capacity for all dyes (*p* < 0.05) (Table 3), confirming a clear dose-dependent response. Importantly, the interaction between dye concentration and species was also significant, indicating that the two diatoms respond differently to increasing dye levels. In contrast, the interactions between species and pH or temperature were not significant (Table 3), indicating that the two species exhibited similar responses to changes in these conditions (Zgłobicka and Kurzydłowski, 2022; Cvjetinovic et al., 2023). Such variability is consistent with adsorption processes governed by electrostatic interactions, molecular size, and structural compatibility between dye molecules and diatom frustules. Across all dyes, *P. tricornutum* exhibited higher adsorption capacity and removal efficiency than *N. veneta* under matched conditions. *P. tricornutum* demonstrated consistently high removal efficiencies, exceeding 80% across most dye types and concentrations (Figures 2–4). Maximum removal was observed for CV at 10 μM, reaching 91.28% ± 0.57%. By contrast, *N. veneta* exhibited lower and more variable removal, particularly at elevated dye concentrations, where BF removal decreased to 56.79% ± 1.17%. This may be attributed to differences in frustule structures, specific surface areas, porosity, or surface functional groups between the two species (Howard et al., 2025; Chuberre et al., 2022; Cantonati et al., 2016), which influence adsorption mechanisms such as electrostatic interactions, hydrogen bonding, and π-π interactions. However, the absence of significant interaction effects for most conditions (Table 3) indicates that environmental factors such as pH and temperature primarily govern adsorption, while species control the baseline adsorption capacity. Both species showed the same temperature-dependent trends, with strong enhancement for MG and BF and little effect for CV, and the temperature × species interaction was not significant. This indicates that temperature effects were governed by dye properties rather than by species. *N. veneta* followed the same trends as *P. tricornutum* but at consistently lower removal. Overall, *P. tricornutum* maintained stable and high removal across a wide pH range, whereas *N. veneta* showed greater sensitivity to acidic conditions, particularly for BF adsorption.

Surface charge may also contribute to the observed difference. A pH_pzc_ of 9.90 ± 0.03 has been reported for *P. tricornutum* (Santaeufemia et al., 2021), although this value was not measured in the present study and no corresponding pH_pzc_ value has been reported for *N. veneta*. By contrast, many other diatoms exhibit much lower pH_*pzc*_ values. For instance, *Nanofrustulum* strains have pH_*pzc*_ values between 5.3 and 6.0 (Golubeva et al., 2023a), and the pH_*pzc*_ values of other diatom-derived biosilica can be as low as 4.9 (Dubey et al., 2024). The stronger pH sensitivity observed for *N. veneta* may reflect differences in surface charge properties and proton-responsive functional groups, potentially associated with a lower pH_pzc_. In addition, heterogeneity in surface charge distribution and frustule microstructure may further contribute to species-specific adsorption behavior. As a marine microalga, *P. tricornutum* is naturally adapted to seawater, which has a high ionic strength. The fact that *P. tricornutum* to maintain strong adsorption under seawater conditions suggests a high affinity for pollutants in high-ionic-strength environments (Santaeufemia et al., 2021), allowing it to better withstand interference from surrounding ions than freshwater species such as *N. veneta*. Lastly, the surface of *P. tricornutum* is enriched with diverse functional groups, as identified by FTIR analysis (Supplementary Figure S2), including hydroxyl (-OH), amide, carboxyl, and carbohydrate-associated groups (Ashour et al., 2022; Howard et al., 2025). These functional moieties provide multiple pathways for pollutant capture, enabling dye adsorption not only through electrostatic interactions but also via cation exchange, π-π interactions, and hydrogen bonding. The two diatom species have distinct structural and chemical properties. Diatom frustules are hierarchically organized three-dimensional architectures that vary significantly across species (Chuberre et al., 2022; Rahman et al., 2024). *N. veneta* possesses a more intricate and highly porous frustule morphology than *P. tricornutum* (Figure 1) (Howard et al., 2025; Cantonati et al., 2016). This structural complexity suggests that its dye removal is diffusion-limited, governed by diffusion-controlled uptake, where pore diffusion or intra-particle diffusion acts as the rate-limiting step as molecules navigate dense rows of areolae to reach internal binding sites. By contrast, the relatively thinner frustule of *P. tricornutum* likely has more accessible surface binding sites, allowing for more rapid equilibrium with less internal mass transfer resistance (Figure 7). However, specific surface area and porosity were not directly measured in this study; therefore, these factors should be interpreted as possible contributors rather than confirmed mechanisms. Future work should include surface area, pore-volume, and pore-size distribution analyses to quantitatively link diatom frustule structure with adsorption performance. Overall, species identity set the baseline adsorption capacity, consistent with the higher adsorption affinity of *P. tricornutum* and the distinct frustule structures of the two species, although the specific contribution of surface area and surface charge remains to be tested.

### 3.5 Limitations and future research

Several aspects of this study could be addressed in future work. First, the equilibrium isotherms were evaluated over a relatively narrow, micromolar dye concentration range, which limited the ability to reach saturation and reliably estimate maximum adsorption capacities for all systems. Consequently, fitted maxima that substantially exceeded the experimentally measured capacities were considered poorly constrained extrapolations rather than physically meaningful adsorption capacities. Future studies incorporating additional equilibrium points over a broader concentration range would better constrain the isotherm parameters and provide more reliable estimates of maximum adsorption capacity. Second, FTIR and SEM-EDS were carried out on the dried biomass before sorption but not afterward. Post-adsorption FTIR would help identify functional groups involved in dye binding, while textural characterization by gas physisorption could determine whether the higher adsorption by *P. tricornutum* is associated with differences in surface area, pore volume, or pore accessibility. A zeta-potential measurement would further test the role of surface charge in dye binding proposed here. HPLC analysis of the supernatant would also help confirm that dye removal reflects biosorption rather than degradation, complementing the abiotic controls used here. Third, the kinetic analysis was based on four time points, which allowed comparison of pseudo-first-order and pseudo-second-order models but was insufficient to resolve successive diffusion regimes in the Weber–Morris analysis. A denser temporal sampling scheme would provide a more robust assessment of the rate-limiting processes. Finally, single dyes were used in DI water to isolate the effects of biomass state and species. However, the performance of diatom biomass in real wastewater, which contains multiple dyes, competing ions, and variable water quality, remains to be verified, ideally using *in-situ* characterization to resolve dye-surface interactions under realistic conditions. Addressing these aspects would strengthen the basis for scaling up diatom-based biosorbents for practical wastewater treatment.

## 4 Conclusion

This study demonstrates that dye adsorption by diatom biomass is governed by the combined effects of solution chemistry (pH and temperature), species-specific structural properties, and physiological states. Adsorption capacity increased with dye concentration because of enhanced mass transfer driving force, while pH and temperature exerted dye-dependent influences. Neutral pH conditions consistently supported high adsorption. Among the tested dyes, CV was insensitive to pH and temperature, indicating contributions from non-electrostatic interactions. By contrast, MG and BF were more responsive to pH and temperature because of changes in ionization and surface charge. Species played a critical role, with *P. tricornutum* consistently outperforming *N. veneta*, achieving higher removal and adsorption capacities across all dyes and conditions tested. Physiological states further controlled adsorption, with dried frustules significantly outperforming live cells. The removal of EPS and membrane barriers during drying is the most plausible explanation for this enhancement, although confirming it requires characterization of the biomass surface before and after adsorption. By contrast, live cells removed less dye than dried biomass under identical conditions. Isotherm analysis indicated adsorption onto an energetically heterogeneous surface, with the Sips model giving the best fit for CV and MG on *N. veneta*, and the Freundlich model performing better for BF. This work demonstrates that dried *P. tricornutum* biomass is an effective biosorbent to remove cationic dyes from wastewater under environmentally relevant conditions. The findings show that diatom biomass is a promising low-cost biosorbent with strong potential for wastewater effluent treatment and value-added biomass utilization.

## Data Availability

The raw data supporting the conclusions of this article will be made available by the authors, without undue reservation.

## References

[B1] Abdullah Al-Dhabi N. Arasu M. V. (2022). Biosorption of hazardous waste from the municipal wastewater by marine algal biomass. Environ. Res. 204:112115. doi: 10.1016/j.envres.2021.112115 34563525

[B2] Al-Ghouti M. A. Khraisheh M. A. M. Allen S. J. Ahmad M. N. (2003). The removal of dyes from textile wastewater: a study of the physical characteristics and adsorption mechanisms of diatomaceous earth. J. Environ. Manage. 69, 229–38. doi: 10.1016/j.jenvman.2003.09.005 14580724

[B3] Al-Tohamy R. Ali S. S. Li F. Okasha K. M. Mahmoud Y. A. G Elsamahy T. . (2022). A critical review on the treatment of dye-containing wastewater: ecotoxicological and health concerns of textile dyes and possible remediation approaches for environmental safety. Ecotoxicol. Environ. Saf. 231:113160. doi: 10.1016/j.ecoenv.2021.113160 35026583

[B4] Arshad H. Imran M. Ashraf M. Naeem A. Riaz M. A. (2025). Removal of structurally different textile dyes from water by adsorption onto biomass of non-viable *Chlorella sorokiniana* strain HIN-3: kinetics and adsorption isotherms. J. Appl. Phycol. 37:973–85. doi: 10.1007/s10811-025-03445-9

[B5] Ashour M. Alprol A. E. Khedawy M. Abualnaja K. M. Mansour A. T. (2022). Equilibrium and kinetic modeling of crystal violet dye adsorption by a marine diatom, *Skeletonema costatum*. Materials 15:6375. doi: 10.3390/ma15186375 36143687 PMC9505319

[B6] Bayramoglu G. Burcu Angi S. Acikgoz-Erkaya I. Yakup Arica M. (2022). Preparation of effective green sorbents using O. Princeps alga biomass with different composition of amine groups: comparison to adsorption performances for removal of a model acid dye. J. Mol. Liq. 347:118375. doi: 10.1016/j.molliq.2021.118375

[B7] Benhalima T. Ferfera-Harrar H. (2026). Fabrication and evaluation of alginate/diatomaceous earth hybrid hydrogels for dual dye adsorption from wastewater. Environ. Sci. Pollut. Res. 33, 3573–93. doi: 10.1007/s11356-026-37509-1 41706348

[B8] Berradi M. Hsissou R. Khudhair M. Assouag M. Cherkaoui O. El Bachiri A. . (2019). Textile finishing dyes and their impact on aquatic environs. Heliyon 5:e02711. doi: 10.1016/j.heliyon.2019.e02711 31840123 PMC6893069

[B9] Bu Z. Fang Y. Chen H. Zhang M. Wang F. (2025). Study on the adsorption properties of organically modified diatomite for methylene blue. Sci. Rep. 15:27561. doi: 10.1038/s41598-025-13768-9 40731074 PMC12307881

[B10] Buhani J.uliasih, N. Wahyu Purnamasari I. Suharso Noviany S.umadi . (2026). Selective adsorption and mechanistic insights into divalent cadmium, copper, and lead recovery using silica-magnetite modified *Nitzschia* sp. biomaterial. Int. J. Phytoremediation 28, 1258–77. doi: 10.1080/15226514.2025.2607515 41548094

[B11] Cantonati M. Angeli N. Spitale D. Lange-Bertalot H. J. F. (2016). A new *Navicula (Bacillariophyta)* species from low-elevation carbonate springs affected by anthropogenic disturbance. Fottea 16, 255–65. doi: 10.5507/fot.2016.013

[B12] Cely Bautista M. M. Cardozo Arrieta B. M. Jaramillo Colpas J. E. Moreno Aldana L. C. Valencia Ríos J. S. Castellar Ortega G. C. (2022). Evaluation of diatomaceous earth in the removal of crystal violet dye in solution. J. Appl. Res. Tech. 20, 387–98. doi: 10.22201/icat.24486736e.2022.20.4.1524

[B13] Chuberre C. Chan P. Walet-Balieu M. L. Thiébert F. Burel C. Hardouin J. . (2022). Comparative proteomic analysis of the diatom *phaeodactylum tricornutum* reveals new insights into intra- and extra-cellular protein contents of its oval, fusiform, and triradiate morphotypes. Front. Plant Sci. 13:673113. doi: 10.3389/fpls.2022.673113 35386671 PMC8977783

[B14] Cvjetinovic J. Luchkin S. Y. Statnik E. S. Davidovich N. A. Somov P. A. Salimon A. I. . (2023). Revealing the static and dynamic nanomechanical properties of diatom frustules—Nature's glass lace. Sci. Rep. 13:5518. doi: 10.1038/s41598-023-31487-x 37015973 PMC10073200

[B15] Dubey S. Mishra R. K. Kaya S. Rene E. R. Giri B. S. Sharma Y. C. (2024). Microalgae derived honeycomb structured mesoporous diatom biosilica for adsorption of malachite green: process optimization and modeling. Chemosphere 355:141696. doi: 10.1016/j.chemosphere.2024.141696 38499077

[B16] Golubeva A. Roychoudhury P. Dabek P. Palczynska J. Pryshchepa O. Piszczek P. . (2023a). A novel effective bio-originated methylene blue adsorbent: the porous biosilica from three marine diatom strains of *Nanofrustulum* spp. (Bacillariophyta). *Sci. Rep*. 13:9168. doi: 10.1038/s41598-023-36408-6 37280270 PMC10244400

[B17] Golubeva A. Roychoudhury P. Dabek P. Pryshchepa O. Pomastowski P. Pałczyńska J. . (2023b). Removal of the basic and diazo dyes from aqueous solution by the frustules of *Halamphora cf. salinicola* (Bacillariophyta). *Marine Drugs* 21:312. doi: 10.3390/md21050312 37233506 PMC10223004

[B18] González Fernández L. A. Castillo Ramos V. Sánchez Polo M. Medellín Castillo N. A. (2023). Fundamentals in applications of algae biomass: a review. J. Environ. Manage. 338:117830. doi: 10.1016/j.jenvman.2023.117830 37004486

[B19] González V. Abalde J. Torres E. (2024). Discoloration and biosorption of Brilliant green dye in seawater using living biomass of the microalga *Phaeodactylum tricornutum*. J. Appl. Phycol. 36:1823–35. doi: 10.1007/s10811-024-03200-6

[B20] Guillard R. R. L. Lorenzen C. J. (1972). Yellow-green algae with chlorophyllide c. J. Phycol. 8, 10–14. doi: 10.1111/j.0022-3646.1972.00010.x

[B21] Howard C. Wang H. Brown J. B. Rao Y. Hou L. (2025). Binding strength and transport kinetics of organic dyes into different live diatoms using second harmonic scattering spectroscopy. ACS Appl. Bio. Mater. 8, 299–309. doi: 10.1021/acsabm.4c01191 39656873

[B22] Islam T. Repon M. R. Islam T. Sarwar Z. Rahman M. M. (2023). Impact of textile dyes on health and ecosystem: a review of structure, causes, and potential solutions. Environ. Sci. Pollut. Res. 30, 9207–42. doi: 10.1007/s11356-022-24398-3 36459315

[B23] Khraisheh M.A. M Al-Ghouti M. A. Allen S. J. Ahmad M. N. (2005). Effect of OH and silanol groups in the removal of dyes from aqueous solution using diatomite. Water Res. 39, 922–32. doi: 10.1016/j.watres.2004.12.008 15743639

[B24] Kowalska J. Jeżewska A. (2019). Determination of pararosaniline hydrochloride in workplace air. Environ. Monit. Assess. 191:444. doi: 10.1007/s10661-019-7568-z 31209660 PMC6579866

[B25] Kumar A. Sidharth S. Kandasubramanian B. (2023). A review on algal biosorbents for heavy metal remediation with different adsorption isotherm models. Environ. Sci. Pollut. Res. 30, 39474–93. doi: 10.1007/s11356-023-25710-5 36780087

[B26] Kumari S. Verma A. Sharma P. Agarwal S. Rajput V. D. Minkina T. . (2023). Introducing machine learning model to response surface methodology for biosorption of methylene blue dye using Triticum aestivum biomass. Sci. Rep. 13:8574. doi: 10.1038/s41598-023-35645-z 37237060 PMC10219957

[B27] Li C. Wang L. Seidi F. Sun W. Gu R. Xiao H. (2021). Naturally occurring exopolysaccharide nanoparticles for dye adsorption. ACS Appl. Nano Mater. 4, 10458–10466. doi: 10.1021/acsanm.1c01978

[B28] Lin J. Ye W. Xie M. Seo D. H. Luo J. Wan Y. . (2023). Environmental impacts and remediation of dye-containing wastewater. Nat. Rev. Earth Environ. 4, 785–803. doi: 10.1038/s43017-023-00489-8

[B29] Long Z. Wang Z. Huang Q. Jia Y. Jiao Z. Wang Y. . (2024). High-performance adsorption of methylene blue using novel bio-adsorbent based on sargassum fusiforme. Heliyon 10:e37949. doi: 10.1016/j.heliyon.2024.e37949 39381252 PMC11458969

[B30] Mani S. Bharagava R. N. (2016). “Exposure to crystal violet, its toxic, genotoxic and carcinogenic effects on environment and its degradation and detoxification for environmental safety,” in: *Reviews of Environmental Contamination and Toxicology*, ed. de Voogt, W. P. (Cham: Springer International Publishing) Volume 237. doi: 10.1007/978-3-319-23573-8_4 26613989

[B31] Mani S. Chowdhary P. Bharagava R. N. (2019). “Textile wastewater dyes: toxicity profile and treatment approaches,” in Emerging and Eco-Friendly Approaches for Waste Management, Bharagava, R. N., and Chowdhary, P. (Singapore: Springer Singapore). doi: 10.1007/978-981-10-8669-4_11

[B32] Ouettar L. Guechi E.-K. Hamdaoui O. Fertikh N. Saoudi F. Alghyamah A. (2023). Biosorption of triphenyl methane dyes (malachite green and crystal violet) from aqueous media by Alfa (Stipa tenacissima L.) leaf powder. Molecules 28:3313. doi: 10.3390/molecules28083313 37110547 PMC10144545

[B33] Prihartini Aryanti P. T. Nugroho F. A. Lugito G. Khoiruddin K. (2025). Tight ultrafiltration membranes: advancing separation technologies for water and wastewater treatment. Sep. Purif. Technol. 367:132892. doi: 10.1016/j.seppur.2025.132892

[B34] Przystaś W. Zabłocka-Godlewska E. Grabińska-Sota E. (2012). Biological removal of azo and triphenylmethane dyes and toxicity of process by-products. Water Air Soil Pollut. 223:1581–92. doi: 10.1007/s11270-011-0966-7 22593606 PMC3332345

[B35] Rahman T. T. Jiang T. Zhang C. Rao Y. Sims R. C. Hou L. (2024). Diatoms in wastewater treatment: potentials, applications, and values of biomass. Crit. Rev. Environ. Sci. Technol. 54:557–80. doi: 10.1080/10643389.2023.2259278

[B36] Rai A. Sirotiya V. Ahirwar A. Singh G. Kawatra R. Sharma A. K. . (2025). Textile dye removal using diatomite nanocomposites: a metagenomic study in photosynthetic microalgae-assisted microbial fuel cells. RSC Adv. 15, 8300–14. doi: 10.1039/D5RA00793C 40103993 PMC11912554

[B37] Saccardo A. Wolf J. Bezzo F. Hankamer B. (2024). Modelling dynamics of photosynthetic units and microalgae growth based on high throughput pulsed light screens. Chem. Eng. J. 490:151684. doi: 10.1016/j.cej.2024.151684

[B38] Santaeufemia S. Abalde J. Torres E. (2021). Efficient removal of dyes from seawater using as biosorbent the dead and living biomass of the microalga Phaeodactylum tricornutum: equilibrium and kinetics studies. J. Appl. Phycol. 33, 3071–90. doi: 10.1007/s10811-021-02513-0

[B39] Shimizu S. Matubayasi N. (2023). Understanding sorption mechanisms directly from isotherms. Langmuir 39, 6113–6125. doi: 10.1021/acs.langmuir.3c00256 37071933 PMC10157891

[B40] Shindhal T. Rakholiya P. Varjani S. Pandey A. Ngo H. H. Guo W. . (2021). A critical review on advances in the practices and perspectives for the treatment of dye industry wastewater. Bioengineered 12, 70–87. doi: 10.1080/21655979.2020.1863034 33356799 PMC8806354

[B41] Srivastava S. Sinha R. Roy D. (2004). Toxicological effects of malachite green. Aqu. Toxicol. 66, 319–29. doi: 10.1016/j.aquatox.2003.09.008 15129773

[B42] Su C. Mu Y. Zhan H. Chen G. Yang K. Fu Q. . (2025). Hierarchical micro–nano porous structure of diatom biosilica induces selective absorption and protease inhibition for bleeding control. ACS Nano 19, 26673–89. doi: 10.1021/acsnano.5c06075 40679442

[B43] Thompson C. O. Ndukwe A. O. Asadu C. O. (2020). Application of activated biomass waste as an adsorbent for the removal of lead (II) ion from wastewater. Emerg. Contam. 6, 259–267.

[B44] Upadhyay R. Khan H. I. U.-H. Przystaś W. (2023). An evaluation of decolorization mechanism of synthetic dyes belonging to the azo, anthraquinone, and triphenylmethane group, as a sustainable approach, by immobilized CB8 strain (Trametes versicolor). Desalination Water Treat. 284, 268–77. doi: 10.5004/dwt.2023.29270

[B45] Utex (2025a). Algal Culture Media F/2 Medium. Available online at: https://utex.org/products/f-2-medium (Accessed July 21, 2025).

[B46] Utex (2025b). Algal Culture Media Modified COMBO Medium. Available online at: https://utex.org/products/modified-combo-medium (Accessed July 21, 2025).

[B47] Wang X. Wang B. Liu Y. Wang J. Cheng J. J. Daroch . (2025). A highly efficient adsorbent adapting to low pH condition for Pb(II) sequestration from aqueous solution – marine diatom: laboratory and pilot scale tests. Sep. Purif. Technol. 353:128321. doi: 10.1016/j.seppur.2024.128321

[B48] Yaseen D. A. Scholz M. (2019). Textile dye wastewater characteristics and constituents of synthetic effluents: a critical review. Int. J. Environ. Sci. Tech. 16, 1193–226. doi: 10.1007/s13762-018-2130-z

[B49] Yilmazer Keskin S. Demir N. Keskin C. S. Avci A. (2025). Bioacumulation and biosorption properties of Penicillium funiculosum against textile dyes. Appl. Water Sci. 15:269. doi: 10.1007/s13201-025-02629-5

[B50] Youssef E. E. Beshay B. Y. Tonbol K. Makled S. O. (2023). Biological activities and biosorption potential of red algae (Corallina officinalis) to remove toxic malachite green dye. Sci. Rep. 13:13836. doi: 10.1038/s41598-023-40667-8 37620415 PMC10449875

[B51] Yuan Y. Li J. Zhang S. Dong C. Tang S. Tu Z. . (2025). Efficient uranium biosorption by marine diatom *Phaeodactylum tricornutum* from simulated seawater: performances and mechanism. J Radioanal. Nucl. Chem. 334, 6335–47. doi: 10.1007/s10967-025-10376-9

[B52] Zgłobicka I. Kurzydłowski K. J. (2022). Multi-length scale characterization of frustule showing highly hierarchal structure in the context of understanding their mechanical properties. Mater. Today Commun. 33:104741. doi: 10.1016/j.mtcomm.2022.104741

