# Supplementary material for: Biosorption of synthetic dyes by live and dried marine diatom Phaeodactylum tricornutum and freshwater diatom Navicula cryptocephala var. veneta
Source: Front Microbiol. 2026 Sep 30;17:1891020. doi: 10.3389/fmicb.2026.1891020 (PMC13662007; doi:10.3389/fmicb.2026.1891020)
Supplement: Supplementary file 1 [file Data_Sheet_1.docx]

**Supplementary Materials**

**Biosorption of Synthetic Dyes by Live and Dried Marine Diatom *Phaeodactylum tricornutum* and Freshwater Diatom *Navicula cryptocephala var. veneta***

**Authors**: Ceaira Howard^1,2^, Tahira Tasneem Rahman^1,2^, Mingyue Li^3^, Kesler Hortin^2^, Chiqian Zhang^4^, Liyuan Hou^1,2, 5^*

**Affiliations**:

^1^Department of Civil and Environmental Engineering, Utah State University, Logan, UT 84322, USA

^2^Utah Water Research Laboratory, 1600 Canyon Road, Logan, UT 84321, United States

^3^School of Resources and Environmental Engineering, Shandong University of Technology, Zibo 255049, China

^4^Civil Engineering Program, College of Engineering & Computer Science, Arkansas State University, Jonesboro, AR 72467, USA

^5^Department of Biological Engineering, Utah State University, Logan, UT 84322, United States

***Correspondence to**: liyuan.hou@usu.edu (Liyuan Hou)

Textbox S1. Calibration curves were established to relate optical density at 680 nm (OD₆₈₀) to dry biomass concentration for each diatom species (Figure S1). Cultures with known biomass concentrations were measured at OD₆₈₀. Linear regression was applied to obtain the following relationships:

*Phaeodactylum tricornutum*:

$$Dry biomass \left( \frac{mg}{L} \right)= 1044 ({OD}_{680})-19.53$$

*Navicula veneta*:

$Dry biomass\left( \frac{mg}{L} \right)= 2,715 ({OD}_{680})-98.56$

**Figure S1.** Calibration curves relating optical density at 680 nm (OD₆₈₀) to dry biomass concentration for *P. tricornutum* and *N. veneta*. Linear regression was used to derive species-specific conversion equations (*R*² = 0.9569 to 0.9617), which were subsequently applied to estimate biomass concentrations in adsorption experiments.

**Figure S2**. FTIR spectra of *N. veneta* (Blue) and *P. tricornutum* (Orange), showing characteristic functional groups including hydroxyl (O–H), amide (I and II), and silanol-related (Si–O–Si) bands contributing to biosorption.

**Figure S3**: Removal efficiency (%) of CV, MG and BF of dry N. veneta (A, C, and E) and P. tricornutum (B, D, and F) under varying initial dye concentrations. CV and MG were tested at 1 to 10 µM and BF at 5 to 20 µM. Experiments were conducted at 20 °C with an initial biomass concentration of 0.2 mg/mL. Error bars represent standard deviations (n = 9).

**Figure S4**. pH-dependent removal efficiency of CV, MG, and BF by dried *N. veneta* (A, C, and E) and *P. tricornutum* (B, D, and F) biomass at an initial biomass concentration of 0.2 mg/mL. Experiments were conducted at 20 °C with initial dye concentrations of 10 µM for CV and MG and 20 µM for BF. Error bars represent standard deviation (*n* = 9).

**Figure S5**. Temperature-dependent removal efficiency of CV, MG, and BF by dried *N. veneta* (A, C, and E) and *P. tricornutum* (B, D, and F). Experiments were conducted at 10, 20, and 30 °C with an initial biomass concentration of 0.2 mg/mL. Initial dye concentrations were 10 µM for CV and MG and 20 µM for BF. Error bars represent standard deviations (*n* = 9).

**Figure S6.** Pseudo-first-order (PFO) and pseudo-second-order (PSO) kinetic model fits for the adsorption of CV, MG, and BF by dried P. tricornutum and N. veneta at 20 °C. Black points show experimental data. The solid red line is the PSO fit and the dashed blue line is the PFO fit. Fitted parameters for both models are given in Table 1.

**Figure S7**. Weber-Morris intraparticle diffusion plots for the adsorption of CV, MG, and BF onto dried *P. tricornutum* and *N. veneta* at 20 °C, unadjusted pH, and 0.2 mg/mL biomass. Lines are single-region linear fits of q_t_ against t^0.5^. Fitted parameters are given in Table 2. The four sampling points do not resolve the successive diffusion regimes indicated by the curvature of the data.
